# Teaching during COVID-19 pandemic in practical laboratory classes of applied biochemistry and pharmacology: A validated fast and simple protocol for detection of SARS-CoV-2 Spike sequences

**DOI:** 10.1371/journal.pone.0266419

**Published:** 2022-04-06

**Authors:** Jessica Gasparello, Chiara Papi, Matteo Zurlo, Lucia Carmela Cosenza, Giulia Breveglieri, Cristina Zuccato, Roberto Gambari, Alessia Finotti

**Affiliations:** 1 Department of Life Sciences and Biotechnology, University of Ferrara, Ferrara, Italy; 2 Interuniversity Consortium for Biotechnology (CIB), Trieste, Italy; University of the Witwatersrand, SOUTH AFRICA

## Abstract

The pandemic caused by the SARS-CoV-2 virus (COVID-19) is still a major health issue. The COVID-19 pandemic has forced the university teaching to consider in high priority the switch from in-presence teaching to remote teaching, including laboratory teaching. While excellent virtual-laboratory teaching has been proposed and turned out to be very useful, the need of a real-laboratory in-presence teaching is still a major need. This study was aimed at presenting a laboratory exercise focusing (a) on a very challenging therapeutic strategy, i.e. SARS-CoV-2 diagnostics, and (b) on technologies that are playing a central role in applied biochemistry and molecular biology, i.e. PCR and RT-PCR. The aims of the practical laboratory were to determine: (a) the possibility to identify SARS-CoV-2 sequences starting from a recombinant plasmid and (b) the possibility to discriminate cells with respect to the expression of SARS-CoV-2 Spike protein. This activity is simple (cell culture, RNA extraction, RT-qPCR are all well-established technologies), fast (starting from isolated and characterized RNA, few hours are just necessary), highly reproducible (therefore easily employed by even untrained students). We suggest that this laboratory practical exercises should be considered for face-to-face teaching especially if the emergency related to the COVID-19 pandemic is maintained. The teaching protocol here described might be considered in order to perform fast but meaningful in-presence teaching, making feasible the division of crowded classes in low-number cohorts of students, allowing the maintenance of the required social distance.

## Introduction

A large number of students engaged in scientific disciplines are expected to be very interested in authentic laboratories experiences in molecular biology classrooms [1, 2]. Accordingly, practical laboratory classes teaching molecular pharmacology approaches employed in the development of therapeutic strategies are of great interest to students attending courses in Biotechnology, Applied Biology, Pharmaceutic and Technology Chemistry, Translational Oncology [2].

This need has been deeply affected by the dramatic pandemic caused by the severe acute respiratory syndrome coronavirus (SARS-CoV-2), responsible for COVID-19 (Corona Virus Disease-2019) [3, 4]. The exponential increase in the number of severe COVID-19 cases and associated deaths has required awareness and proactive actions with respect to diagnosis and possible therapeutic treatments [5, 6]. In addition, COVID-19 pandemic was primarily responsible for dramatic changes in the teaching at all levels, including teaching at university [7–10].

The COVID-19 pandemic is still a major health problem worldwide [11, 12]. Only recently, the availability of vaccines and the activation of programs for extensive vaccination contribute to an improvement of the health and social situation [13–16], but we expect that the difficulty in organizing teaching activities will persist. In this respect, we are experiencing dramatic differences in tackling SARS-CoV-2 (for instance with the vaccination campaign), due to the relevant heterogeneity between high-income and low-income countries [17–19]. Therefore, some countries will not be able to solve in short time the teaching difficulties, due to persistence of high levels of SARS-CoV-2 spreading.

In this respect, the COVID-19 pandemic has forced the university teaching to consider in high priority the switch from in-presence teaching to remote teaching [20–22]. This has occurred in the total courses in some universities and high-schools; only recently a partial and strictly controlled rescue was implemented to in-presence teaching [23].

As far as the laboratory teaching, we have experienced the production of lessons focusing on virtual laboratory classes, as proposed by several reports [24–27]. These laboratories facilitate learning of technologies requiring complex instruments, costly reagents and materials and highly trained personnel [24–27].

While excellent virtual-laboratory teaching has been proposed and turned out to be very useful, however the need of a real-laboratory in-presence teaching is still a major need. Unfortunately, in most cases, the technology to be transferred to learning student laboratory classes is complex and requires multi-step approaches [28–30]. In this respect, and in particular during the COVID-19 pandemic, simple and straightforward experimental protocols might be of great interest. In this case, student’s expectation can be met by organizing wet-labs for acquiring complex skills and the ability to discuss challenging biomedical approaches [31–36]. This can be facilitated by hosting real-laboratory in-presence teachings which will be repeated to student in sub-groups avoiding overcrowding and maintaining a social distance during the COVID-19 pandemic [36, 37].

This study was aimed at presenting a laboratory exercise focusing (a) on a very challenging therapeutic strategy, i.e. SARS-CoV-2 diagnostics, and (b) on technologies that are playing a central role in applied biochemistry and molecular biology, i.e. PCR and RT-PCR.

The aims of the practical laboratory were to determine: (a) the possibility to identify SARS-CoV-2 sequences starting from a recombinant plasmid and (b) the possibility to discriminate cells with respect to the expression of SARS-CoV-2 Spike protein.

**Fig 1:**
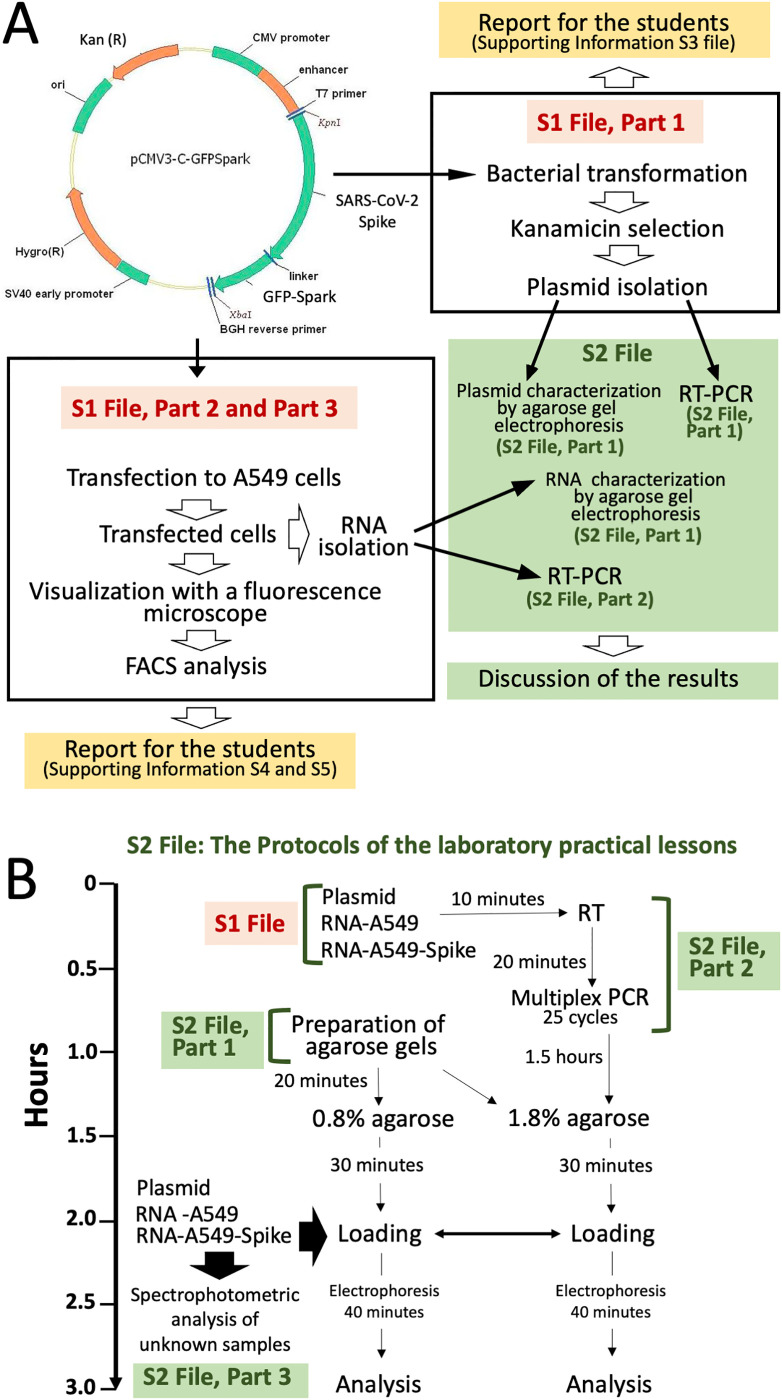
Timing of the laboratory practice. A. Preliminary activity conducted in the absence of students. B. Timing of the practical laboratory showing all the activities to be followed by the students. The protocols are described in S1 and S2 Files. The methodologies are also described in S3–S5 Files.

This practical activity is simple, as culturing of eukaryotic cells, extraction of DNA and RNA, polymerase-chain reaction (PCR) are all well-established technologies available in most of the cellular and molecular biology laboratories. In addition, the results are obtained quickly (in fact, starting from isolated and characterized RNA, few hours are just necessary) and are highly reproducible, allowing the proposed protocols to be employed by even untrained students. The scheme of the laboratory lesson is depicted in Fig 1. It should be underlined that these laboratory lessons require no complex facilities, the most critical being the availability of instruments for PCR, which is anyway a very common instrument in research activities performed in the molecular biology field. On the contrary, the determination of the presence or the lack of amplified products can be obtained using low-cost standard analytical approaches based on agarose gel electrophoresis.

## Materials and methods

### Materials

The lists of the materials are presented in the S1 and S2 Files. In the S1 File (Title: The Protocols for preparing the biological materials for the laboratory practical lessons) the activity is presented for preparing the biological material to be used by the students during the practical laboratory lessons. The protocols presented in the S2 File (Title: The Protocols of the laboratory practical lessons) are related to the sections focusing on the practical activity performed by the students. While the complete lists of the materials are included in the S1 and S2 Files, the following section summarizes the key materials employed. The two protocols have been submitted to the protocol.io platform with the following DOIs: dx.doi.org/10.17504/protocols.io.x54v9y26zg3e/v1 (S1 File) and dx.doi.org/10.17504/protocols.io.bp2l612qzvqe/v1 (S2 File).

#### Plasmid and cell line

1. pCMV3-Spike-GFPSpark^®^ plasmid (SinoBiological, Beijing, China; CMV, CytoMegaloVirus; GFP, Green Fluorescence Protein).
2. A549 human lung carcinoma cell line [38] (ATCC, CCL-185).

#### Cell culture

1. Dulbecco’s Modified Eagle Medium (D-MEM) (Gibco, Thermo Fisher Scientific, Walthman, Massachusetts, USA).
2. 100 U/mL penicillin and 100 μg/mL streptomycin (Sigma-Aldrich, St.Louis, Missouri, USA).
3. Fetal bovine serum (FBS, Biowest, Nauillè, France).
4. Non-Essential Amino Acids Solution 100X (NEAA, Gibco, Thermo Fisher Scientific, Walthman, Massachusetts, USA).
5. To determine cell growth a Z2 Coulter Counter (Coulter Electronics, Hialeah, Florida, USA).

#### Cell transfection

1. Lipofectamine LTX + Plus Reagent (Thermo Fisher Scientific, Walthman, Massachusetts, USA).
2. Opti-MEM-I Reduced Serum Medium (Gibco, Thermo Fisher Scientific, Walthman, Massachusetts, USA).

#### RNA extraction

1. Trypsin-(ethylenedinitrilo)tetraacetate (EDTA) (Sigma-Aldrich, St.Loius Missouri, USA).
2. Dulbecco’s phosphate-buffered saline (DPBS) (Gibco, Thermo Fisher Scientific, Walthman, Massachusetts, USA).
3. Tri-Reagent (Sigma-Aldrich, St.Loius Missouri, USA) was employed for cell lysis.
4. Extracted RNA was quantified using DeNovix DS-11 Spectrophotometer (DeNovix, Wilmington, Delaware, USA).
5. The quality of the RNA was determined by spectrophotometric analysis and by agarose gel electrophoresis.

#### Reverse transcription reaction

1. Reverse transcriptase (RT) reactions were performed using PrimeScript RT Reagent Kit (Perfect Real Time) (Takara, Shiga, Japan) using a mixture of random 6 mers sequences and oligo dT as primers and GeneAmp PCR System 9700 (Thermo Fisher Scientific, Walthman, Massachusetts, USA).

#### Reverse transcription (RT) multiplex PCR

1. Primers for multiplex PCR analysis have been designed using PrimerQuest Tool IDT (Integrated DNA Technology, Newark, New Jersey, USA) and synthesized by IDT. Sequences of employed primers has been reported in Table 1.
2. All RT-PCR reactions were conducted using Wonder Taq Polymerase and its relative buffer: 5X Wonder Taq Buffer (Euroclone, Pero, Milano, Italy). PCR was performed in a final volume of 30 μl using GeneAmp PCR System 9700 (Thermo Fisher Scientific, Walthman, Massachusetts, USA).
3. PCR products were verified by checking on 1.8% (w/v) agarose gel.

**Table 1:** Sequences of primers employed. All primers were designed using PrimerQuest Tool (IDT) and purchased from IDT. Lyophilized primers were resuspended in DNAse/RNAse free water (Sigma-Aldrich) at a stock concentration of 1 μgr/μl, a further dilution was performed before the PCR preparation reaching a primers final concentration of 50 ng/μl, in nuclease-free water.

Primer	Sequence	Amplicon length
SARS-CoV-2 FW	5’-AAGGACCTTCCTGCTGAAATAC-3’	400 bp
SARS-CoV-2 RW	5’-GCAATCTGTCTCACCTCATCTC-3’	
β-actin FW	5’-TCCTCCTGAGCGCAAGTA-3’	700 bp
β-actin RW	5’-GTCTCAAGTCAGTGTACAGGTAAG-3’	

#### Check on agarose gel

1. Tris Acetate EDTA (TAE) 1x (40 mM Tris, 20 mM acetic acid, 1 mM EDTA) was homemade starting from single reagents (Sigma-Aldrich, St.Louis, Missouri, USA).
2. High purity biotechnology grade agarose (Norgen Biotek Corp, Thorold, Ontario, Canada).
3. FluoroVue Nucleic Acid Gel Stain 10.000x (SMOBIO Technology, Sinchu City, Taiwan, ROC).
4. Orange Loading Dye 6X (Thermo Fisher Scientific, Walthman, Massachusetts, USA)
5. GeneRuler 50 bp DNA Ladder (Thermo Fisher Scientific, Walthman, Massachusetts, USA).
6. GeneRuler 1 kb DNA Ladder (Thermo Fisher Scientific, Walthman, Massachusetts, USA).
7. Power Supply and electrophoresis cells (Bio-Rad, Hercules, CA, USA).
8. GelDoc System (Bio-Rad, Hercules, CA, USA).

### Methods

The step-by-step Protocols are presented in S1 and S2 Files, focusing on preparing the biological material to be used by the students during the practical laboratory lessons (the S1 File) and on the practical activity performed by the students (S2 File). The contents of the S1 and S2 Files are also presented in Fig 1. While full details of the methods are included in the S1 and S2 Files, the following section summarizes the key methodological activities, also presented in Fig 1.

#### Human cell lines, culture conditions and transfection

A549 cells were cultured in humidified atmosphere of 5% CO_2_/air in culture medium composed by RPMI-1640 (Sigma-Aldrich) and 10% FBS (Biowest) supplemented with 100 units/mL penicillin and 100 g/mL streptomycin. Cells were seeded at 50% of confluence 12 hours before the transfection. The day after, the medium was removed and replaced with new one. A mixture containing 500 ng of pCMV3-Spike-GFPSpark plasmid (SinoBiological), Lipofectamine LTX Reagent and PLUS Reagent (Thermo Fisher Scientific) was prepared to reach the final volume of 50 μL with Opti-MEM medium (Thermo Fisher Scientific). The mixture was incubated at room temperature (RT) for 30 min to allow the complexation of plasmid DNA to liposomes and then transferred to cells. Cells were maintained in contact with the mixture for 24 h, then plasmid internalization and GFP production were verified. The extraction of the pCMV3-Spike-GFPSpark plasmid is also summarized in the S3 File; the procedures for the transfection of A549 cells with the pCMV3-Spike-GFPSpark plasmid are also summarized in the S4 File.

#### GFP production by A549-Spike cells

In order to verify transfection efficiency, GFP-expressing cells were visualized under a fluorescence microscope (Nikon Eclipse, Nikon Corporation, Minato, Tokyo, Japan) using FITC (fluorescein isothiocyanate) filter, for a qualitative analysis of transfected cells. Moreover, the percentage of GPF positive cells was determined by FACS analysis using FACS Canto II instrument (BD, Becton, Dickinson, Franklin Lakes, New Jersey, USA). Briefly, cells were detached using trypsin-EDTA solution (Sigma-Aldrich), washed twice with DPBS (Gibco) and resuspended in 200 μl of DPBS. 30.000 events were acquired and analysed using FITC channel and BD FACSDiva Software (BD) for data elaboration. The procedures for the characterization of the A549 cells transfected with the pCMV3-Spike-GFPSpark plasmid are described in the S4 File.

#### Total RNA extraction

Cells were trypsinized and collected by centrifugation at 1500 RPM for 10 min at 4°C, washed with DPBS and lysed with Tri-Reagent (Sigma-Aldrich), according to manufacturer’s instructions. The isolated RNA was washed once, with cold 75% ethanol, dried and dissolved in nuclease-free water (Sigma-Aldrich) before use. The RNA was stored at -80°C until the use. The quality of the RNA was determined by spectrophotometric analysis and by agarose gel electrophoresis. Ratio 260/280 and 260/230 were used for determining the overall quality. The electrophoresis on 0,8% agarose in TAE (Tris-acetate-EDTA) buffer was employed for quality checking. The procedures for RNA extraction are also summarized in the S5 File.

#### mRNA reverse transcription

The total RNA was quantified using DeNovix DS-11 Spectrophotometer (DeNovix) and 300 ng of total RNA were reverse transcribed using PrimeScript RT Reagent Kit (Perfect Real Time) (Takara). A mixture (final volume 20 μl) containing random 6 mers sequences and oligo dT as primers, Prime Script RT Enzyme Mix (including reverse transcriptase enzyme and Ribonuclease inhibitor), total RNA and 5X Prime Script Buffer was prepared and incubated at 37°C for 15 min and then at 85°C for 5 sec (enzyme inactivation step) according to the manufacturer’s protocol. The obtained cDNA was stored at -20°C until the use.

#### Multiplex PCR using plasmid DNA or cDNA derived from A549 and A549-Spike cells

One μL of obtained cDNA was amplified in 30 μL (final volume) of RT-PCR reaction mix, containing 5X Wonder Taq Buffer, two primers pairs (50 ng/μl, stock concentration) for the amplification of human β-actin transcript and SARS-CoV-2 spike protein coding sequence (the sequences of the primers are reported in Table 1) and 1 μl of Wonder Taq Polymerase. The following amplification program was employed: 95°C for 3 min (initial denaturation and polymerase activation), 95°C for 15 sec (denaturation phase), 60°C for 30 sec (annealing), 72°C for 30 sec (elongation); steps 2, 3 and 4 were repeated for 25 cycles, at the end a final elongation phase of 72°C for 5 min was added. The obtained PCR products were stored at -20°C until checking on agarose gel was performed.

#### Check on agarose gel

Check on agarose gel was employed for both a) nucleic acids analysis: RNA and plasmid DNA were checked on agarose gel to verify their quality (% of agarose 0.8% w/v) and b) verify PCR products obtained by multiplex PCR (% of agarose 1.8% w/v). Briefly, agarose powder (Norgen Biotek) was dissolved in TAE 1x buffer. When the solution cool down to about 50 °C, FluoroVue Nucleic Acid Gel Stain 10.000x (SMOBIO) was added and the solution was placed into a gel tray with the wells comb in place. 700 ng of unknown nucleic acid (RNA or plasmid) and 10 μl of PCR product were added to Orange Loading Dye 6X (Thermo Fisher Scientific) and nuclease-free water, to reach the final volume of 12 μl. Samples were loaded into the gel and the gel was run at 80V for 45 min. GeneRuler 50 bp DNA Ladder (Thermo Fisher Scientific) and GeneRuler 1 kb DNA Ladder (Thermo Fisher Scientific) were used as molecular weight markers.

## Results

### Preparation of the pCMV3-Spike-GFPSpark plasmid and transfected A549 cells

In order to minimize the length of time of the practical laboratory, some preparative steps were performed with the objective of preparing the following biological material: (a) pCMV3-Spike-GFPSpark plasmid (Spike-plasmid), (b) RNA from A549 cells and (c) RNA from A549 cells transfected with the pCMV3-Spike-GFPSpark plasmid and expressing Spike mRNA and protein (A549-Spike). These activities are in the present laboratory protocol included in preliminary sample preparations and are summarized in S3–S5 Files. These activities should be presented to the students and discussed in streaming in order to minimize the time necessary for the experimental activity performed in presence. However, in the case COVID-19 related restrictions are abandoned, these activities can be part of the practical laboratory lesson and performed by the students under the supervision of the teachers. The protocols concerning these activities are included in the S1 File.

The preparation of the pCMV3-Spike-GFPSpark plasmid (Spike-plasmid) includes bacterial transformation, kanamycin selection, large cultures of transformed bacteria, and plasmid isolation. The steps necessary to produce the Spike plasmid are described in the S3 File. We have produced 125 μg of pCMV3-Spike-GFPSpark plasmid, resuspended in 100 μl of TE (Tris EDTA) buffer (final stock concentration 1250 ng/μl).

The preparation of the RNA from A549 cells and A549 cells expressing Spike was based on transfection of A549 cells with the pCMV3-Spike-GFPSpark plasmid, selection with hygromycin, and characterization of the transfected cells by FACS analysis. The steps necessary to produce and characterize the A549-Spike cells are described in the S4 File. We have produced 10 μg of RNA from the two cell populations, resuspended in 50 μl of RNAse free water (see the S5 File).

The produced plasmid and RNA samples from A549 and A549-Spike cells were sufficient for the programmed laboratory activity of at least 200 students. The white boxes of Fig 1 describe these preliminary activities performed for the preparation of the biological materials.

### Outline of the practical laboratory program

The green insert of Fig 1A reports a summary of the practical laboratory. Fig 1B describes in detail the student’s activity, which includes (a) preparation of the agarose gel; (b) analysis of plasmid and/or RNA samples from A549 and A549-Spike cells; (c) RT-PCR and (d) gel electrophoresis analysis of RT-PCR products. Fig 1B also reports the estimated length of time necessary for performing all these activities.

The outline of the main practical laboratory, starting from the isolated plasmid and RNA samples will answer the following questions:

- What the quality of the pCMV3-Spike-GFPSpark plasmid preparation was?
- What the quality of RNA from A549 and A549-Spike cells was?
- Is the multiplex PCR reaction efficient?
- The obtained RT-PCR products were those expected on the basis of the starting material employed (plasmid, RNA from A549 cells and RNA from A549-Spike cells)?

### Discussion with the students of the characterization of the A549 and A549-Spike cells and the expected results of the quality control to be performed during their practical activity

**Fig 2:**
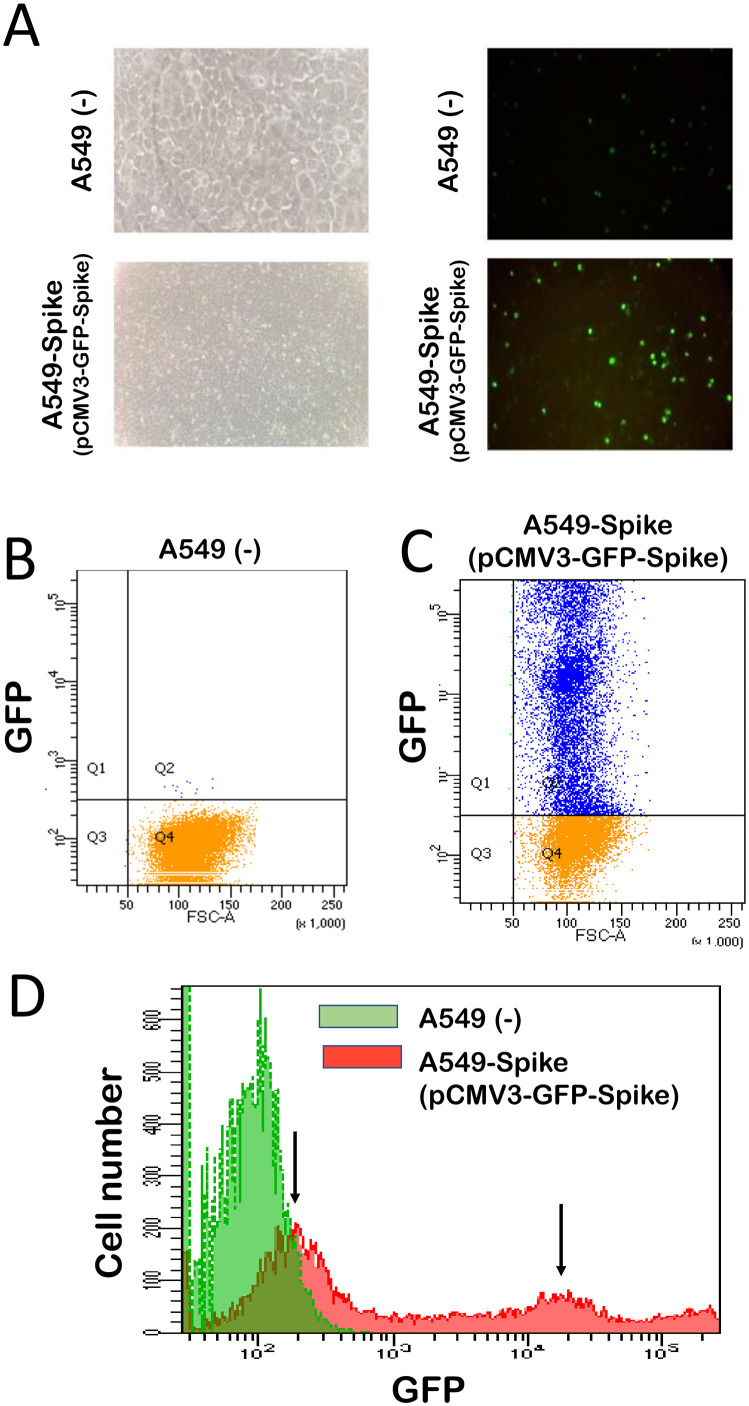
Preliminary activity (see Fig 1A): Characterization of untreated A549 cells and A549 cells transfected with the pCMV3-GFP-Spike plasmid. A. Microscopy-based analysis. On the bottom-right side of this panel a fluorescence analysis is shown demonstrating high number of fluorescent A549-Spike cells. B,C. FACS analysis of untreated (B) and pCMV3-GFP-Spike plasmid treated (C) A549 cells. D. Densitometry analysis of the FACS profiles shown in B and C, demonstrating that all the A549-Spike cells are fluorescent, but at least two populations are present, identified by black arrows (red histogram).

Fig 2 shows a characterization of the A549 and A549-Spike cells. Cells have been analyzed by fluorescence microscopy (Fig 2A) and by cytofluorometry (Fig 2B and 2C). Fig 2A shows that A549 cells display very low or absent fluorescence activity. On the contrary, A549 cells exhibit high fluorescence activity (due to GFP produced following transfection with the pCMV3-Spike-GFPSpark plasmid), which was found in at least 70–80% of the cell population. This information was fully confirmed by the FACS analysis shown in Fig 2B–2D. No GFP-positive cells were present in control un-transfected A549 cells (Fig 2B and green histogram of Fig 2D). On the contrary, the majority of the A549-Spike cells were GFP-positive (Fig 2C and red histogram of Fig 2D), despite with different levels of GFP expression.

**Fig 3:**
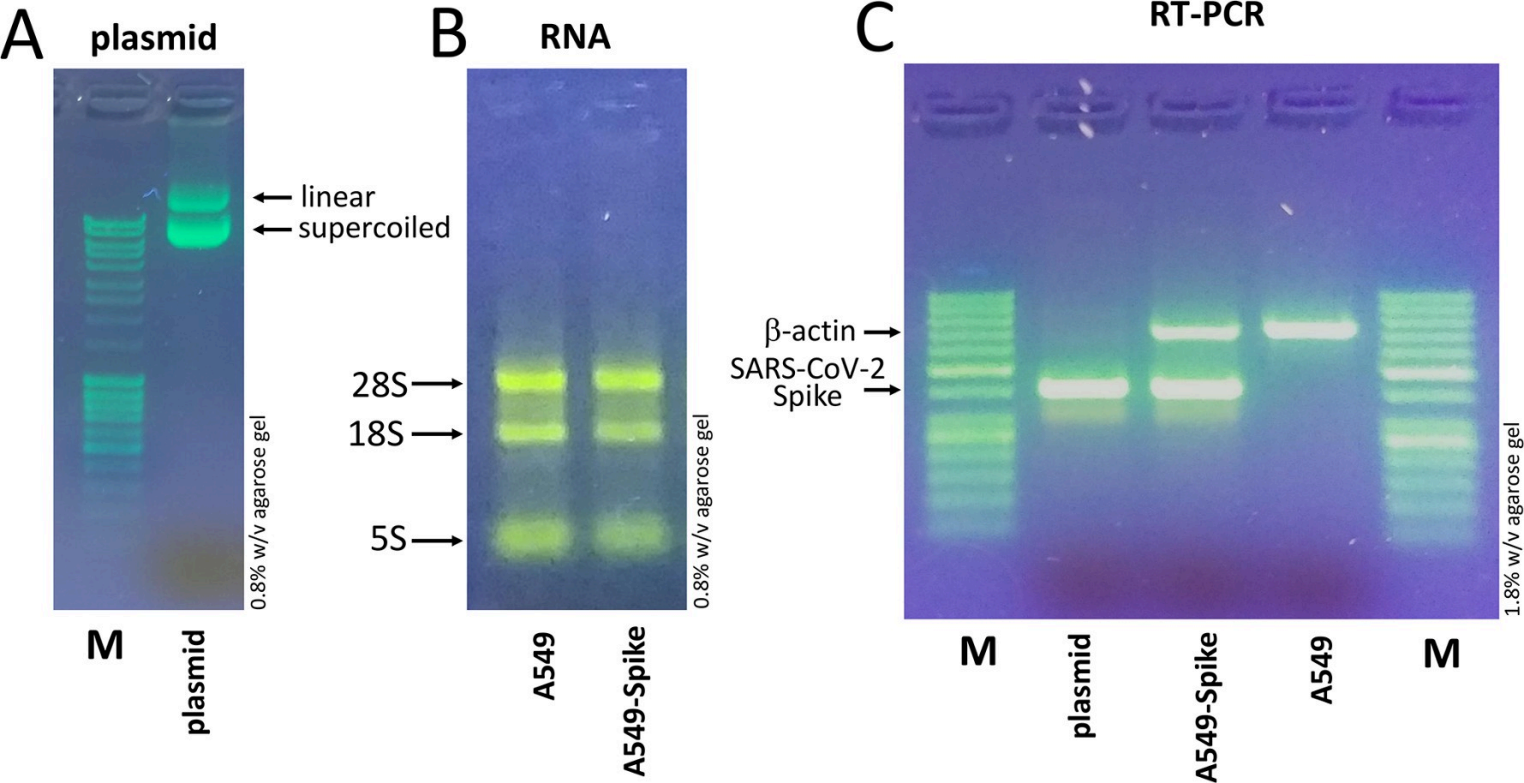
Preliminary activity (see Fig 1A): Characterization of the pCMV3-GFP-Spike plasmid and the RNA produced by untreated A549 cells and A549 cells transfected with the pCMV3-GFP-Spike plasmid. A. 0.8% w/v agarose gel electrophoresis of the pCMV3-GFP-Spike plasmid. B. 0.8% w/v agarose gel electrophoresis of RNA isolated from untreated A549 cells or cells transfected with the pCMV3-GFP-Spike plasmid. C. Check on 1.8% w/v agarose gel of amplicons obtained with Multiplex PCR done using the pCMV3-GFP-Spike plasmid, or cDNA using RNA isolated from un-transfected A549 cells, or A549 cells transfected with the pCMV3-GFP-Spike plasmid. The primers employed for PCR are able to co-amplifying, in the same reaction, β-actin and Spike-SARS-CoV-2 sequences. The raw uncropped images are included in the S6 File.

The quality control of the pCMV3-Spike-GFPSpark plasmid and the RNA from control A549 cells and A549-Spike cells is shown in Fig 3A and 3B, respectively. The students will be informed that linear and supercoiled forms of the pCMV3-Spike-GFPSpark plasmid are expected (see Fig 3A). As far as RNA, the analysis will be judged corrected if 28S, 18S and 5S RNAs will be visible and clearly separated (see Fig 3B).

The second part of the laboratory practical activity was to perform an RT-reaction and a multiplex PCR analysis using primers co-amplifying β-actin and Spike-SARS-CoV-2 sequences. The primers have been optimized before the student’s activity and found excellent in originating the following results, depending on the starting material used. The expected results obtained by the RT-multiplex-PCR analysis are shown in Fig 3C. When the pCMV3-Spike-GFPSpark plasmid is employed, only amplification of Spike-SARS-CoV-2 sequences should be detected (400 bp amplicon size). In this case, it is important to point out with the students that cDNA production did not occur during the reverse transcription and therefore the template of the multiplex PCR reaction was the Spike coding plasmid itself.

Accordingly, when RNA from control A549 cells is employed only amplification of β-actin sequences should be detected (700 bp amplicon size); finally, when RNA from A549-Spike cells is employed both β-actin and Spike-SARS-CoV-2 sequences should be detected, as these cells have been elsewhere demonstrated to be able to express the plasmid (see Fig 2) and therefore the Spike-GFP mRNA.

The ratio of the β-actin versus Spike-SARS-CoV-2 amplified sequences might be different depending on the A549-Spike population employed for the preparation of the RNA sample; however, the same RNA preparation was given to the class to which this protocol was carried out.

It might be intriguing to discuss with the students the parallelism between the results obtained with the plasmid, the A549 and A549-Spike derived amplicons with respect to the development of COVID-19 diagnostic assays allowing discrimination between genomic viral positive control and SARS-CoV-2 positive samples.

### The organization of the practical laboratory: General procedures

In the process of admission of the students we kept the required records of who attended including their physical proximity (e.g., lab bench numbers). The students have been divided in 6 groups never exceeding 16 students for each group. The time that students spent in the lab was kept at the minimum as this action reduces the opportunities for SARS-CoV-2 transmission. This was facilitated by the design of the practical exercise (see Fig 1B) that needed no more than 3 hours to be completed, allowing classes to be divided into multiple sequential streams, also facilitating an effective cleaning of the used laboratories.

Students have been fully prepared before coming to the laboratory class, in order to use the time just for the practical activity. Pre-lab workflow plans and presentation of the scientific materials included in Supporting Information files were used. The learning objectives for laboratory activities have been precisely described.

General safety procedures [39–41] were followed. For example, body temperature measurements were taken and hand washing/sanitation was done both on entry and exit as well as at regular intervals. In addition, cleaned lab clothes, face masks and gloves (when appropriate) were used. All the surfaces have been decontaminated by the staff before starting of the practical laboratory activity, by the students when they arrived at and left the workstation. A final decontamination of the used surfaces has been done by the staff after students left the laboratory.

The overall period of teaching was two weeks (3 days/week). The 81 students were divided in 6 working groups (Group 1: n = 16; Group 2: n = 14; Group 3: n = 14; Group 4: n = 9; Group 5: n = 13; Group 6: n = 15). Each group was programmed to work for a total of 3 hours, according to Fig 1B. Each group was responsible for preparing two agarose gels (0.8% and 1.8% agarose) for electrophoresis to be performed for the part concerning quality control of plasmid DNA and RNAs from A549 and A549-Spike cells.

### The results of the practical laboratory: Part 1

**Fig 4:**
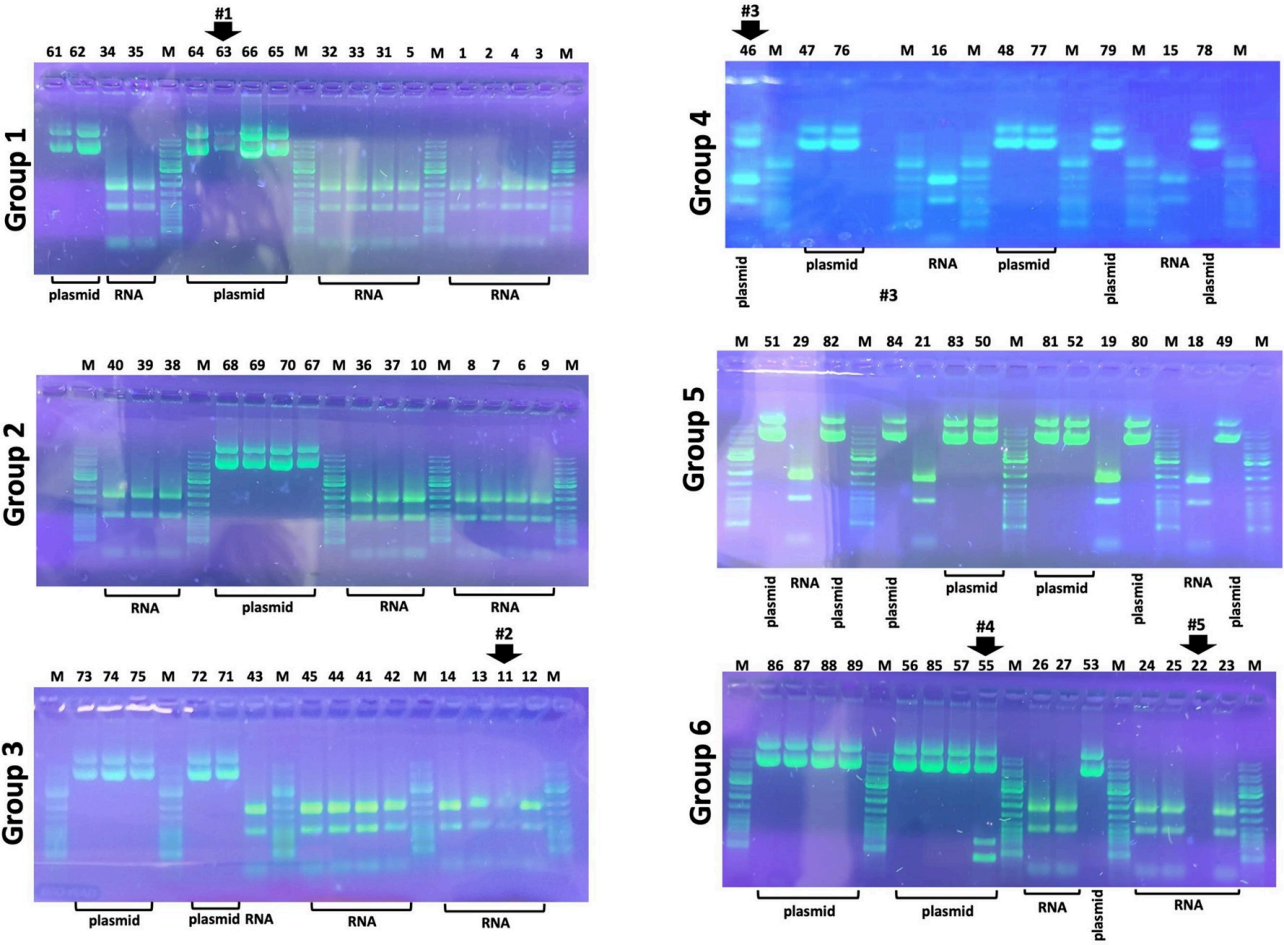
Gel electrophoretic analysis of the pCMV3-GFP-Spike plasmid and the RNA produced by A549 cells. The analysis was conducted by the participating 89 students with excellent results, with the exception of students 63 (Group 1), 11 (Group 3), 46 (Group 4), 22 and 55 (Group 6). The raw uncropped images are included in the S6 File.

The results of the quality control determinations performed by the students are shown in Fig 4, which shows the analysis performed using the 0.8% agarose gels, according to the procedure outlined in Fig 1B. Each student was responsible for loading 10 μl of an unknown sample (RNA or plasmid). Loading errors were found in three cases, indicating no loading in one case (arrow #5), loading of lower amount of material in two cases (arrows #1 and #2). Two students contaminated the plasmid DNA preparations, in one case with RNA samples (arrows #3), in the other case with RT-PCR samples obtained using a template RNA from A549 (arrow #4). In total, 5 errors over 81 attempts (6.17%).

### The results of the practical laboratory: Part 2

**Fig 5:**
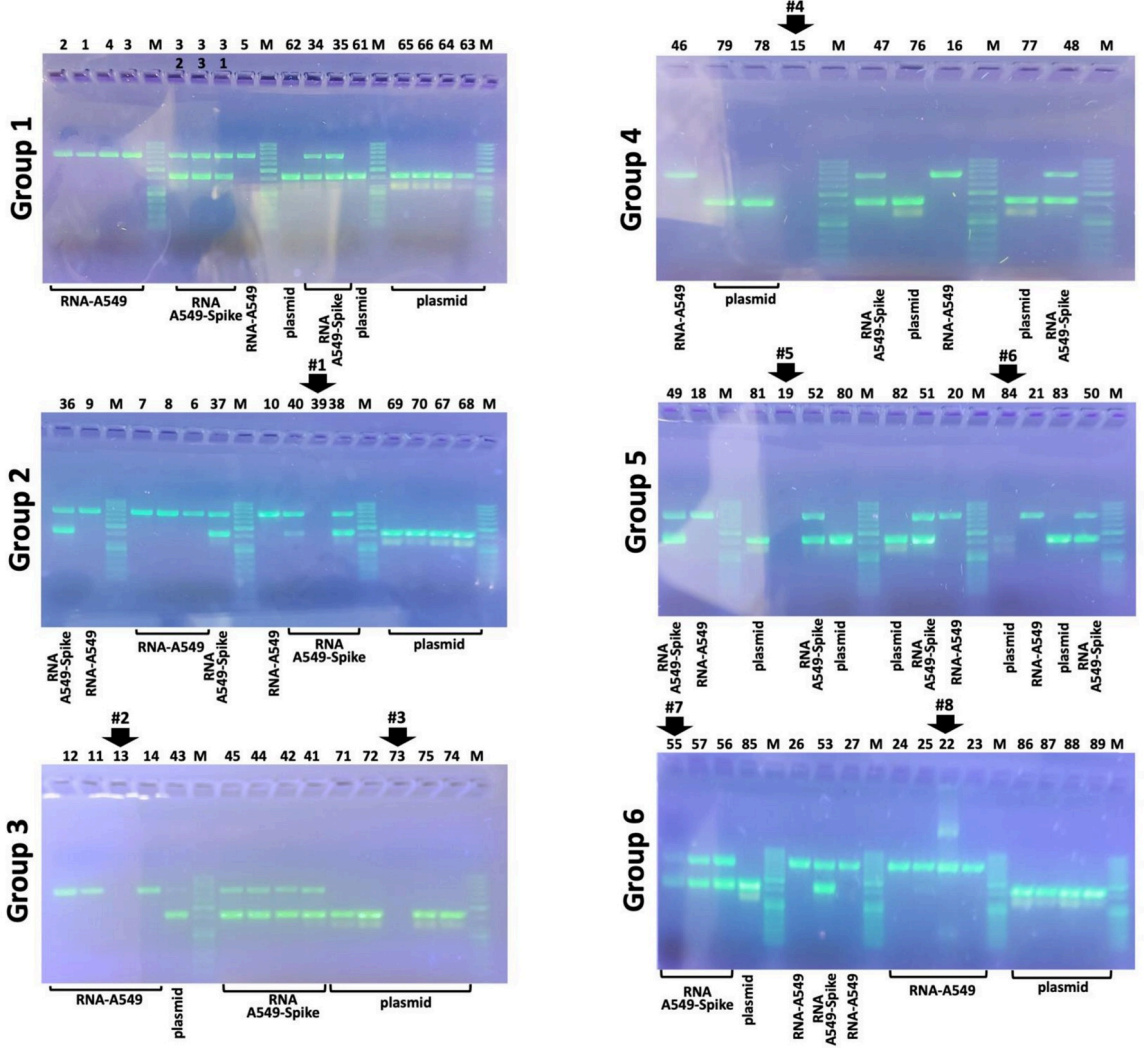
Multiplex PCR amplifying Spike SARS-CoV-2 and β-actin sequences and performed on pCMV3-GFP-Spike plasmid, and cDNA obtained using RNA isolated from untreated A549 cells or A549 cells transfected with the pCMV3-GFP-Spike plasmid, as indicated. The analysis was conducted by the participating 89 students with good results, except for students 39 (Group 1), 13 and 73 (Group 3), 15 (Group 4), 19 and 84 (Group 5), 22 and 55 (Group 6). Notably, students 22 and 55 failed to obtain positive results also when performing gel electrophoretic analysis of the pCMV3-GFP-Spike plasmid and the RNA produced by A549 cells (see Fig 4). The raw uncropped images are included in the S6 File.

The results of the RT-PCR analysis using plasmid DNA, RNA from A549 cells and RNA performed by the students are shown in Fig 5, which shows the analysis performed using the 1.8% agarose gels, according to with the procedure outlined in Fig 1B. Each student was responsible for loading 10 μl of PCR products derived by an RT reaction starting from (a) plasmid DNA, (b) RNA from A549 cells and (c) RNA from A549-Spike cells. Loading errors were found in 7 cases, indicating no loading in 5 cases (arrows #1- #5), loading of lower amount of material in two cases (arrows #6 and #7). In one case (arrow #8) in addition to the expected band corresponding to RT-PCR product, two additional extra bands were visualized.

## Discussion

It is globally recognized that higher education institutions are highly vulnerable to community transmission of the SARS-CoV-2 virus and increased number of COVID-19 infected students and staff. For this reason, almost all universities suspended face-to-face academic activities during COVID-19 pandemic, implementing alternative ways of teaching [42–45].

One of the strategies adopted by many universities was the switching of the teaching activity to online delivery [46]. Virtual labs, remote control labs or video-based labs are good choices when students are not physically located on campus [47]. For virtual labs, simulation tools and virtual reality are used [48]. Remote laboratories allow the undertaking of experiments through the internet, whereas video-based activities provide a step-by-step overview of a real lab so that students can visualize the whole experimental process and its environment through a video [48–53].

Simple experiments answering to key issues in applied pharmacology could be of great interest in the teaching, with particular focus on the possibility to set up practical exercises in laboratory practice delivered to the students in the field of biotechnology, pharmaceutics and applied biology.

In this manuscript, we present simple experiments that can be the basis for laboratory practical teaching with the aim of determining: (a) the quality of preparation of plasmid DNA to be used in transfection procedures; (b) the quality of RNA from control cells and cells transfected with a recombinant plasmid; (c) the efficiency and reproducibility of a multiplex PCR reaction; (d) the possible differences between expected and obtained results of the multiplex PCR. As model systems, we used the pCMV3-Spike-GFPSpark plasmid and the A549 cell line. The multiplex PCR was performed using primers optimized for the co-amplification of SARS-CoV-2 Spike and β-actin sequences in the same reaction.

The results which can be obtained during this laboratory teaching activity support the following conclusions. First of all, the large majority of the students were able to assess the quality of pCMV3-GFP-Spike plasmid and the quality of RNA produced by untreated A549 cells and A549 cells transfected with the pCMV3-GFP-Spike plasmid (compare the data of Fig 4 with those presented in Fig 3, panels A and B). Second, in the multiplex RT-PCR performed by the large majority of the students, the data obtained were those expected (compare the data of Fig 5 with those presented in Fig 3C).

A key point of the proposed practical activity is its simplicity (in fact cell culture methodologies, RNA extraction procedure and RT-PCR technologies are all well-established in higher schools and universities). The practical lesson needs to be completed of a short period of time (in fact, starting from isolated and characterized RNA, only few hours are necessary). Finally, the techniques employed and the reagents developed lead to highly reproducible results (enabling the involvement of even untrained students). On the other hand, some facilities are necessary, the most critical of which being instruments for PCR. For this reason we propose the use of simple and low-cost PCR instruments and the analysis of the amplified products by standard approaches based on agarose gel electrophoresis. These PCR instruments and proposed methodologies are commonly available in most of molecular biology laboratories.

We suggest that this laboratory practical exercise should be considered for face-to-face teaching especially if the emergency related to the COVID-19 pandemic is maintained [11, 12]. This is due to the fact that there is very strong evidence for the importance of laboratory courses, especially in the scientific field [54].

While the SARS-COV-2 vaccination has improved the overall situation related to the COVID-19 pandemic [14–16], the solutions followed by different countries to limit viral spread within high schools and university is highly heterogenous. In addition, we cannot exclude the necessity of repeated lock-down strategies, especially considering SARS-CoV-2 variants [55]. Finally, we are experiencing dramatic differences in the anti-SARS-CoV-2 vaccination campaign when high-income and low-income countries are compared [17–19].

## Conclusions

The teaching protocols here described (and detailed in S1 and S2 Files) might be considered in order to perform simple and reproducible in-presence teaching focusing on a very challenging therapeutic strategy (i.e. SARS-CoV-2 diagnostics) and on technologies that are playing a central role in applied biochemistry and molecular biology, (i.e. PCR and RT-PCR). The practical laboratory protocols were efficient in identifying SARS-CoV-2 sequences starting from a recombinant plasmid and discriminating cells with respect to the expression of SARS-CoV-2 Spike protein. The teaching protcols are fast (few hours are just necessary to complete the practical lessons), making feasible the division of crowded classes in low-number cohorts of students, allowing the maintenance of the required social distance.

## Supporting information

S1 File The protocols for preparing the laboratory practical lessons. (PDF)

S2 File The protocols of the laboratory practical lessons. (PDF)

S3 File Extraction of the pCMV3-C-GFPSpark plasmid DNA. (PDF)

S4 File Transfection of A549 cells with pCMV3-C-GFPSpark plasmid. (PDF)

S5 File RNA extraction. (PDF)

S6 File Raw images. (PDF)
