# Supplementary material for: Teaching during COVID-19 pandemic in practical laboratory classes of applied biochemistry and pharmacology: A validated fast and simple protocol for detection of SARS-CoV-2 Spike sequences
Source: PLoS One. 2022 Apr 6;17(4):e0266419. doi: 10.1371/journal.pone.0266419 (PMC8985952; doi:10.1371/journal.pone.0266419)
Supplement: S1 File — (PDF) [file pone.0266419.s001.pdf]

S1 File The Protocols for preparing the laboratory practical lessons

protocol

Apr 12, 2022

PLOS One

Jessica Gasparello[1], Matteo Zurlo[1], Lucia Carmela Cosenza[1], Giulia Breveglieri[1], Cristina Zuccato[1], Roberto Gambari[1,2], Alessia Finotti[1]

[1]Department of Life Sciences and Biotechnology University of Ferrara 44121 Ferrara Italy;

[2]Interuniversity Consortium for Biotechnology (CIB) 34012 Trieste Italy

1

dx.doi.org/10.17504/protocols.io.b6perdje

PLOS ONE Lab Protocols
Tech. support email: **plosone@plos.org**

gam

The protocols here described are related to the sections presented in Figure 1A (open boxes) focusing on the activity performed in preparing the biological material to be used by the students during the practical laboratory lessons. All the steps are presented and critically commented when deemed useful. The reagents used during the preparation of these protocols are indicated as an example, as all the reagents can be purchased from any company.

DOI

dx.doi.org/10.17504/protocols.io.b6perdje

https://doi.org/10.1371/journal.pone.0266419

Jessica Gasparello, Matteo Zurlo, Lucia Carmela Cosenza, Giulia Breveglieri, Cristina Zuccato, Roberto Gambari, Alessia Finotti 2022. S1 File The Protocols for preparing the laboratory practical lessons. **protocols.io** https://dx.doi.org/10.17504/protocols.io.b6perdje

protocol

Gasparello J, Papi C, Zurlo M, Cosenza LC, Breveglieri G, et al. (2022) Teaching during COVID-19 pandemic in practical laboratory classes of applied biochemistry and pharmacology: A validated fast and simple protocol for detection of SARS-CoV-2 Spike sequences. PLOS ONE 17(4): e0266419. https://doi.org/10.1371/journal.pone.0266419

Citation: Jessica Gasparello, Matteo Zurlo, Lucia Carmela Cosenza, Giulia Breveglieri, Cristina Zuccato, Roberto Gambari, Alessia Finotti S1 File The Protocols for preparing the laboratory practical lessons https://dx.doi.org/10.17504/protocols.io.b6perdje

This is an open access protocol distributed under the terms of the **Creative Commons Attribution License** (https://creativecommons.org/licenses/by/4.0/), which permits unrestricted use, distribution, and reproduction in any medium,

protocol ,

Mar 24, 2022

Mar 24, 2022

59846

S1 File The Protocols for preparing the laboratory practical lessons

1 A549 cell culture and transfection with pCMV3-Spike-GFPSpark plasmid

1.1 Thaw bacteria (*E.coli* JM109 strain) on ice for about ten minutes (use about 100 µl aliquots);

1.2 Mix bacteria by finger vortexing and add 10 ng of pCMV-C-GFPSpark by slowly pipetting (no more than twice);

1.3 Incubate on ice for 30 min and then at 42°C for 1 min

1.4 Put on ice for 2 min

1.5 Add 200 µl of LB (Luria-Bertani)-medium (Triptone; Yeast Extract; NaCl) without antibiotic

1.6 Incubate at 37°C for 1 hour, keeping in slow shaking (about 90 rpm);

1.7 Incubate at 37°C for 1 hour, keeping in slow shaking (about 90 rpm);

Citation: Jessica Gasparello, Matteo Zurlo, Lucia Carmela Cosenza, Giulia Breveglieri, Cristina Zuccato, Roberto Gambari, Alessia Finotti S1 File The Protocols for preparing the laboratory practical lessons https://dx.doi.org/10.17504/protocols.io.b6perdje

This is an open access protocol distributed under the terms of the **Creative Commons Attribution License** (https://creativecommons.org/licenses/by/4.0/), which permits unrestricted use, distribution, and reproduction in any medium,

1.8 The day after pick a colony and grow bacteria carrying the pCMV-GFP plasmid in 100 ml of LB-medium (Luria Bertani medium) with antibiotic (Kanamycin, final concentration 25 µg/ml) at 37°C under shaking until the desired density is reached

1.9 Take about 50 ml of culture medium containing bacteria grown up to the plateau (turbid medium); centrifuge at 3,000 rpm for 30 min at 4°C; remove the supernatant by inverting the 50 ml tube

1.10 Resuspend the pellet in 5 ml of buffer P1 added with 100 µg/ml RNase A and Lyse Blue solution

1.11 Add 4 ml of buffer P2, shake by inversion for 4 times, until the solution became blue or light blue, incubate 5 min at RT

1.12 Add 5 ml of buffer P3, shake by inversion 4 times (comment: white precipitated particles will form) incubate on ice for 15 min

1.13 Centrifuge for 30 min at 4°C, 3,000 rpm; take the supernatant (containing the plasmid DNA)

1.14 Equilibrate a QIAGEN-tip adding 4 ml of QBT buffer, allow column to become empty by gravity flow, then add the supernatant and allow it to enter the resin by gravity flow

1.15 Wash the column 2 times with 10 ml of QC Buffer, allowing the buffer to move through the QIAGEN-tip by gravity flow

1.16 Elute the plasmid with 5 ml of QF buffer into a clean tube

1.17 Elute the plasmid with 5 ml of QF buffer into a clean tube

Centrifuge 1.5 ml tubes at 12,000 rpm for 30 min at 4°C, discard the

Citation: Jessica Gasparello, Matteo Zurlo, Lucia Carmela Cosenza, Giulia Breveglieri, Cristina Zuccato, Roberto Gambari, Alessia Finotti S1 File The Protocols for preparing the laboratory practical lessons https://dx.doi.org/10.17504/protocols.io.b6perdje

This is an open access protocol distributed under the terms of the **Creative Commons Attribution License** (https://creativecommons.org/licenses/by/4.0/), which permits unrestricted use, distribution, and reproduction in any medium,

1.18 supernatant by inverting 1.5 ml tubes and dry tubes using a lab paper

1.19 Add 400 µl of 75% EtOH to each tube and centrifuge at 12,000 rpm 4°C for 10 minutes

1.20 Remove the ethanol and allow the pellets to dry in the air, under the hood

1.21 Resuspend the plasmid DNA pellet in TE (Tris-EDTA pH = 8)

2 A549 cell culture and transfection with pCMV3-Spike-GFPSpark plasmid

2.1 Detach A549 cells, when are at confluence, from a starting T25 flask. Briefly: remove cell supernatant, wash the cells once with 5 ml of DPBS, add 1 ml of trypsin-EDTA and incubate at 37°C for 5 min. Add 1 ml of FBS to inactivate trypsin action and add 8 ml of DMEM to restore the starting volume

2.2 Plate A549 cells in a 12-well plate at 35-40% confluence one day before the transfection using DMEM + FBS 10% as medium

2.3 Incubate cells for 24 hours at 37°C, 5% CO_2

2.4 The day after, remove the old medium, and replace it with 1 ml of new medium composed by DMEM + FBS 10%. The procedure should be performed at least 30 min before the transfection. At the day of the transfection cells must be at 70-80% of confluence;

2.5 Prepare a mixture containing 1 µg of pCMV3-Spike-GFPSpark plasmid, 1 µl of PLUS Reagent and 47.5 µl of opti-MEM. Incubate the mixture at RT for 5 min

Add 2.5 µl of Lipofectamine LTX to the mixture and incubate at room

Citation: Jessica Gasparello, Matteo Zurlo, Lucia Carmela Cosenza, Giulia Breveglieri, Cristina Zuccato, Roberto Gambari, Alessia Finotti S1 File The Protocols for preparing the laboratory practical lessons https://dx.doi.org/10.17504/protocols.io.b6perdje

This is an open access protocol distributed under the terms of the **Creative Commons Attribution License** (https://creativecommons.org/licenses/by/4.0/), which permits unrestricted use, distribution, and reproduction in any medium,

2.6 temperature for 30 min

2.7 Add 2.5 µl of Lipofectamine LTX to the mixture and incubate at room temperature for 30 min

2.8 Stir the plate and incubate at 37°C, 5% $CO_2$for 24 hours

2.9 The day after observe cells at fluorescence microscope using FITC filter (Excitation 490, Emission 525);

2.10 Remove the supernatant, wash the well with 1 ml of DPBS, add 200 µl of trypsin and incubate at 37°C for 5 min, add the same volume (200 µl) of FBS, restore the starting well-volume with 600 µl of DMEM

2.11 Centrifuge detached cells at 1,200 rpm for 8 min RT

2.12 Centrifuge detached cells at 1,200 rpm for 8 min RT

2.13 Centrifuge again as indicated at point “k”

2.14 Remove the supernatant and resuspend the pellet by adding 300 µl of DPBS

2.15 Remove the supernatant and resuspend the pellet by adding 300 µl of DPBS

3 RNA extraction.

Citation: Jessica Gasparello, Matteo Zurlo, Lucia Carmela Cosenza, Giulia Breveglieri, Cristina Zuccato, Roberto Gambari, Alessia Finotti S1 File The Protocols for preparing the laboratory practical lessons https://dx.doi.org/10.17504/protocols.io.b6perdje

This is an open access protocol distributed under the terms of the **Creative Commons Attribution License** (https://creativecommons.org/licenses/by/4.0/), which permits unrestricted use, distribution, and reproduction in any medium,

3.1 Detach cells adding 200 µl of trypsin to each well, incubate for 5 min at 37°C, add the same volume of FBS to inactivate trypsin and restore the starting well volume with 600 µl of DMEM

3.2 Collect cells and centrifuge at 1,200 rpm for 8 min at RT

3.3 Remove supernatant and wash the cellular pellet with DPBS

3.4 Lyse cellular pellets adding 900 µl of TRI Reagent under a chemical hood: immediately after addition, pipette quickly to make the system homogeneous (comment: no white fragments of the pellet should be present)

3.5 Incubate the samples for 5 min at room temperature (RT), keeping them in the rack

3.6 Add 200 µl of RNase-free chloroform, under a chemical hood. (Comment: chloroform is very volatile and tends to drip; to avoid this, prime the tip before taking the chloroform);

3.7 Mix vigorously by inversion for 15 sec, until the solution becomes homogeneous and the two phases can no longer be distinguished

3.8 Incubate the samples at RT for 2-3 min, keeping them in the rack

3.9 Centrifuge at 12,000 rpm, for 15 min, at 4 ° C. The mixture separates into 3 phases:
-Upper aqueous phase: transparent, contains RNA
-Whitish semi-solid interface: contains DNA and proteins
-Lower organic phase: pink, contains DNA, proteins and lipids

3.10 Take the upper aqueous phase without touching or drawing the interface or the pink organic phase, and transfer it into a new 1.5 ml RNase-free tube: work under a chemical hood

Citation: Jessica Gasparello, Matteo Zurlo, Lucia Carmela Cosenza, Giulia Breveglieri, Cristina Zuccato, Roberto Gambari, Alessia Finotti S1 File The Protocols for preparing the laboratory practical lessons https://dx.doi.org/10.17504/protocols.io.b6perdje

This is an open access protocol distributed under the terms of the **Creative Commons Attribution License** (https://creativecommons.org/licenses/by/4.0/), which permits unrestricted use, distribution, and reproduction in any medium,

3.11 Add 500 µl of 100% RNase-free isopropanol to precipitate the RNA and mix well by inverting the 1.5 ml tube. (Comment: Isopropanol is very volatile and can drip, to avoid this, prime the tip before taking the isopropanol);

3.12 Incubate the samples at RT for 10 min

3.13 Centrifuge at 12,000 rpm, for 15 min, at 4°C. A pellet containing the RNA will be obtained

3.14 Discard the supernatant with the pipette

3.15 Add 900 µl of 75% RNase-free ethanol, pre-cooled on ice, (Comment: the ethanol is very volatile and can drip, to avoid this, prime the tip before taking the solution)

3.16 Gently invert the 1.5 ml tubes to wash the pellets. Observe carefully the size of the pellet obtained, this is essential to establish the volume of water to be used to resuspend the pellet

3.17 Centrifuge at 12,000 rpm, for 5 min, at 4°C

3.18 Discard the supernatant with the pipette

3.19 Allow the RNA pellets to dry in the air under the hood for about 10 min

3.20 Resuspend the RNA in 20 µl or more of RNase-free H_2O, pipetting several times (Comment: the volume of water to be added may vary based on the size of the pellet and will be established after careful observation of the pellet)

Citation: Jessica Gasparello, Matteo Zurlo, Lucia Carmela Cosenza, Giulia Breveglieri, Cristina Zuccato, Roberto Gambari, Alessia Finotti S1 File The Protocols for preparing the laboratory practical lessons https://dx.doi.org/10.17504/protocols.io.b6perdje

This is an open access protocol distributed under the terms of the **Creative Commons Attribution License** (https://creativecommons.org/licenses/by/4.0/), which permits unrestricted use, distribution, and reproduction in any medium,

3.21 Store on ice, if analyzed immediately, or freeze at -80°C

Citation: Jessica Gasparello, Matteo Zurlo, Lucia Carmela Cosenza, Giulia Breveglieri, Cristina Zuccato, Roberto Gambari, Alessia Finotti S1 File The Protocols for preparing the laboratory practical lessons https://dx.doi.org/10.17504/protocols.io.b6perdje

This is an open access protocol distributed under the terms of the **Creative Commons Attribution License** (https://creativecommons.org/licenses/by/4.0/), which permits unrestricted use, distribution, and reproduction in any medium,
