# Supplementary material for: Teaching during COVID-19 pandemic in practical laboratory classes of applied biochemistry and pharmacology: A validated fast and simple protocol for detection of SARS-CoV-2 Spike sequences
Source: PLoS One. 2022 Apr 6;17(4):e0266419. doi: 10.1371/journal.pone.0266419 (PMC8985952; doi:10.1371/journal.pone.0266419)
Supplement: S2 File — (PDF) [file pone.0266419.s002.pdf]

Apr 12, 2022

S2 File The Protocols of the laboratory practical lessons

PLOS One

Jessica Gasparello[1], Chiara Papi[1], Matteo Zurlo[1], Lucia Carmela Cosenza[1], Giulia Breveglieri[1], Cristina Zuccato[1], Roberto Gambari[1,2], Alessia Finotti[1]

[1]Department of Life Sciences and Biotechnology University of Ferrara 44121 Ferrara Italy;

[2]2Interuniversity Consortium for Biotechnology (CIB) 34012 Trieste Italy

1

dx.doi.org/10.17504/protocols.io.b6pgrdjw

PLOS ONE Lab Protocols
Tech. support email: **plosone@plos.org**

Jessica Gasparello

The protocols here described are related to the sections presented in Figure 1B focusing on the practical activity performed by the students. All the steps are presented and critically commented when deemed useful. The reagents used during the preparation of these protocols are indicated as just an example, as all the reagents can be purchased from any company.

DOI

dx.doi.org/10.17504/protocols.io.b6pgrdjw

https://doi.org/10.1371/journal.pone.0266419

Jessica Gasparello, Chiara Papi, Matteo Zurlo, Lucia Carmela Cosenza, Giulia Breveglieri, Cristina Zuccato, Roberto Gambari, Alessia Finotti 2022. S2 File The Protocols of the laboratory practical lessons. **protocols.io**
https://dx.doi.org/10.17504/protocols.io.b6pgrdjw

protocol

Gasparello J, Papi C, Zurlo M, Cosenza LC, Breveglieri G, et al. (2022) Teaching during COVID-19 pandemic in practical laboratory classes of applied biochemistry and pharmacology: A validated fast and simple protocol for detection of SARS-CoV-2 Spike sequences. PLOS ONE 17(4): e0266419. https://doi.org/10.1371/journal.pone.0266419

protocol ,

Citation: Jessica Gasparello, Chiara Papi, Matteo Zurlo, Lucia Carmela Cosenza, Giulia Breveglieri, Cristina Zuccato, Roberto Gambari, Alessia Finotti S2 File The Protocols of the laboratory practical lessons https://dx.doi.org/10.17504/protocols.io.b6pgrdjw

This is an open access protocol distributed under the terms of the **Creative Commons Attribution License** (https://creativecommons.org/licenses/by/4.0/), which permits unrestricted use, distribution, and reproduction in any medium,

Mar 24, 2022

Mar 24, 2022

59848

(a) Agarose powder. High purity biotechnology grade agarose (Norgen Biotek Corp, Thorold, Ontario, Canada);
(b) Tris Acetate EDTA (TAE) 50X. TAE 50X (2 M Tris-base, 1 M acetic acid, 50 mM EDTA disodium salt) was prepared as follow: weigh 242 grams of Tris-base (MW=121.14 g/mol); dissolve Tris-base in 500 ml of deionized water; add, under a chemical hood 57.1 ml s of acetic acid; add 100 ml of 0.5 M EDTA pH 8.0; adjust the volume to a final volume of 1 L. All starting reagents were from Sigma-Aldrich (St.Louis, Missouri, USA);
(c) Nuclease free water;
(d) Tartrazine or other tracking dye (Sigma-Aldrich, St.Louis, Missouri, USA);
(e) Deionized water;
(f) FluoroVue Nucleic Acid Gel Stain 10.000x (SMOBIO Technology, Sinchu City,Taiwan, ROC);
(g) Orange Loading Dye 6X (Thermo Fisher Scientific, Walthman, Massachusetts, USA);
(h) GeneRuler 1 kb DNA Ladder (Thermo Fisher Scientific, Walthman, Massachusetts, USA), as molecular weight marker for 'nucleic acid (RNA or DNA) samples' (optional, we do not use it in our experience) or GeneRuler 50 bp DNA Ladder (Thermo Fisher Scientific, Walthman, Massachusetts, USA) for PCR product molecular weight marker (see 2.1 point);
(i) Unknown sample (more detailed information are provided in supplementary methods for laboratory experience organizers). In any case, the concentration of (i) the pCMV3-Spike-GFPSpark plasmid should be to about 300 ng/µl in our case (we recommended to load not less than 500 ng) and (ii) RNA extracted from A549 and Spike-plasmid transfected A549 cells, also in this case we used 300 ng/µl of RNA.

Part 1: Quality control of (i) the pCMV3-Spike-GFPSpark plasmid (Spike plasmid) and (ii) RNA extracted from A549 and Spike-plasmid transfected A549 cells

1 In a graduated cylinder prepare **1 L** of TAE (Tris-Acetate-EDTA) buffer, 1x **Contributed by users** solution diluting TAE (TRIS-ACETATE-EDTA) Buffer, 50x **Amresco Catalog #K915** stock solution; with deionized water: **20 mL** of TAE 50x and **980 mL** of deionized water are required

2 Prepare the tray to add agarose solution, adding two **1 mm** well combs

Citation: Jessica Gasparello, Chiara Papi, Matteo Zurlo, Lucia Carmela Cosenza, Giulia Breveglieri, Cristina Zuccato, Roberto Gambari, Alessia Finotti S2 File The Protocols of the laboratory practical lessons https://dx.doi.org/10.17504/protocols.io.b6pgrdjw

This is an open access protocol distributed under the terms of the **Creative Commons Attribution License** (https://creativecommons.org/licenses/by/4.0/), which permits unrestricted use, distribution, and reproduction in any medium,

3 Weight 0.8 g of Agarose Contributed by users Catalog #A5304 powder and add it into a glass flask

4 Add to the glass flask 100 mL of TAE (Tris-Acetate-EDTA) buffer, 1x Contributed by users

5 Boil the suspension in a microwave for same minutes, until the solution will be completely clear

6 Add 10 µL of FluoroVue 10000x nucleic acid gel stain and stir the solution until it will be completely homogeneous (agarose solution will become light yellow)

7 Add slowly the agarose solution to the gel tray and carefully remove possible bubbles

8 Let the agarose solution solidify for 00:30:00 at room temperature 30m

9 In the meantime, prepare the unknown sample adding a standard volume of sample: 2.5 µL (Comment: this volume corresponds to about 700 ng of plasmidic DNA or RNA), 2 µL of tartrazine tracking dye and 7.5 µL of nuclease free water. Mix the sample by pipetting three or four times

10 Spin the sample to disrupt possible bubbles and keep the sample on ice until the loading

11 When the gel is solidified, setup the electrophoresis apparatus: remove the tray and set the gel into the electrophoresis chamber, add TAE 1x solution to the electrophoresis chamber, carefully remove 1 mm-well combs

12 Load 10 µL of unknow sample to the gel

Citation: Jessica Gasparello, Chiara Papi, Matteo Zurlo, Lucia Carmela Cosenza, Giulia Breveglieri, Cristina Zuccato, Roberto Gambari, Alessia Finotti S2 File The Protocols of the laboratory practical lessons https://dx.doi.org/10.17504/protocols.io.b6pgrdjw

This is an open access protocol distributed under the terms of the **Creative Commons Attribution License** (https://creativecommons.org/licenses/by/4.0/), which permits unrestricted use, distribution, and reproduction in any medium,

13 Run the agarose gel with TAE 1x buffer, at 80V for 00:40:00 40m

14 Check the results at UV-transilluminator

Part 2: RT-qPCR analysis of (a) plasmid DNA, (b) RNA from A549 cells and (c) RNA from A549-Spike cells

15 In a graduated cylinder prepare 1 L of TAE 1x solution diluting TAE 50x stock solution with deionized water: 20 mL of TAE 50x and 980 mL of deionized water are required

16 Prepare the tray to add agarose solution, adding two 1 mm well combs

17 Weight 1.8 g of agarose powder and add it into a glass flask

18 Add to the glass flask 100 mL of TAE 1x

19 Boil the suspension in a microwave for same minutes, until the solution will be completely clear

20 Add 10 µL of FluoroVue 10000x nucleic acid gel stain and stir the solution until it will be completely homogeneous (agarose solution will become light yellow)

21 Add slowly the agarose solution to the gel tray and carefully remove possible bubbles

22 Let the agarose solution solidify for 00:30:00 at room temperature; in the meantime, 30m

Citation: Jessica Gasparello, Chiara Papi, Matteo Zurlo, Lucia Carmela Cosenza, Giulia Breveglieri, Cristina Zuccato, Roberto Gambari, Alessia Finotti S2 File The Protocols of the laboratory practical lessons https://dx.doi.org/10.17504/protocols.io.b6pgrdjw

This is an open access protocol distributed under the terms of the **Creative Commons Attribution License** (https://creativecommons.org/licenses/by/4.0/), which permits unrestricted use, distribution, and reproduction in any medium,

prepare the sample adding 2 µL of Orange loading dye to 10 µL of PCR product

23 Spin the sample to disrupt possible bubbles and keep the sample on ice until the loading

24 When the gel is solidified, setup the electrophoresis apparatus: remove the tray and set the gel into the electrophoresis chamber, add TAE 1x solution to the electrophoresis chamber, carefully remove 1 mm -well combs

25 Load 10 µL of sample to the gel or 5 5 µL of Gene Ruler 50 bp

26 Check the gel at UV-transilluminator

Reverse transcription

27 Thaw the unknown samples and spin to put the sample at the bottom of the 0.2 mL tube. Keep the sample on ice for all the experience

28 Dilute the sample with 10 µL of nuclease free water

29 Add 4 µL 5x Prime Script Buffer and the same volume of Random 6 mers [M]100 Micromolar (µM)

30 Add 1 µL of Oligo dT primer [M]50 Micromolar (µM)

31 Add 1 µL of Prime Script RT Enzyme Mix 1. The final volume of 20 µL will be reached. (Comment: enzyme must be stored in a refrigerated block, to avoid repeated freeze and thaw cycles)

Citation: Jessica Gasparello, Chiara Papi, Matteo Zurlo, Lucia Carmela Cosenza, Giulia Breveglieri, Cristina Zuccato, Roberto Gambari, Alessia Finotti S2 File The Protocols of the laboratory practical lessons https://dx.doi.org/10.17504/protocols.io.b6pgrdjw

This is an open access protocol distributed under the terms of the **Creative Commons Attribution License** (https://creativecommons.org/licenses/by/4.0/), which permits unrestricted use, distribution, and reproduction in any medium,

32 Pipette up and down three or four times with a p20 pipette set at 20 µL

33 Spin down the sample with a microcentrifuge

34 20m

Incubate the sample within a thermal cycler set at 37°C for 00:15:00 for complementary DNA synthesis, then incubate at 85 °C for 00:05:00 to denature the reverse transcriptase enzyme. After the thermic program the sample must keep on ice. (Note this is a stop point, if required sample can be store at -20 °C for several days).
(Comment: during the thermic program of the reverse transcription, the preparation of PCR sample will be started).

RT-PCR

35 Add at the bottom of 0.2 ml tube 16 µL of nuclease free water

36 Add 1.5 µL of each primers concentrated 50 ng/µl

37 Carefully add 1 µL of DNA generated at point 1 and keep on ice

38 Add 1 µL of Wonder Taq Polymerase. The final volume of 30 µL will be reached. (Note for organizers and students: enzyme must be stored in a refrigerated block, to avoid repeated freeze and thaw cycles)

39 Pipette up and down three or four times with a p200 pipette set at 30 µL 3

40 Spin down the sample with a microcentrifuge

Citation: Jessica Gasparello, Chiara Papi, Matteo Zurlo, Lucia Carmela Cosenza, Giulia Breveglieri, Cristina Zuccato, Roberto Gambari, Alessia Finotti S2 File The Protocols of the laboratory practical lessons https://dx.doi.org/10.17504/protocols.io.b6pgrdjw

This is an open access protocol distributed under the terms of the **Creative Commons Attribution License** (https://creativecommons.org/licenses/by/4.0/), which permits unrestricted use, distribution, and reproduction in any medium,

41 Incubate the sample within a thermal cycler setting the following amplification program: 4m 15s

- 95 °C 00:03:00 initial denaturation and polymerase activation)
- 95 °C 00:00:15 denaturation phase)
- 60 °C 00:00:30 annealing)
- 72 °C 00:00:30 elongation)

-Steps 2, 3 and 4 were repeated for 25 cycles

42 At the end a final elongation phase of 72 °C for 00:05:00 min was added. After the thermic program the sample must keep on ice. (Comment: this is a stop point, if required sample can be store at -20°C for several days) 5m

43 10 µl of obtained PCR products, will be loaded on the [M]1.8 Mass / % volume agarose gel

Part 3: Quantitative control of (a) the pCMV3-Spike-GFPSpark plasmid (Spike plasmid) and (b) RNA extracted from A549 and Spike-plasmid transfected A549 cells

44 Thaw the sample and pipette up and down two or three times to mix the sample

45 Add 1 µL of sample diluent (in our case nuclease free water) onto the optical sensor and click 'read blank'

46 Clear the optical sensor with deionized water

47 Withdraw 1 µL of the sample and add directly onto the optical sensor

Citation: Jessica Gasparello, Chiara Papi, Matteo Zurlo, Lucia Carmela Cosenza, Giulia Breveglieri, Cristina Zuccato, Roberto Gambari, Alessia Finotti S2 File The Protocols of the laboratory practical lessons https://dx.doi.org/10.17504/protocols.io.b6pgrdjw

This is an open access protocol distributed under the terms of the **Creative Commons Attribution License** (https://creativecommons.org/licenses/by/4.0/), which permits unrestricted use, distribution, and reproduction in any medium,

48 Click ‘read sample’ and note the two absorbance values: A_{260} and A_{280}

49 After [M] **0.8 Mass / % volume** agarose gel visualization calculate sample concentration considering that:

plasmidic DNA: 1OD of absorbance at 260 nm correspond to 50 µg/ml; RNA: 1OD of absorbance at 260 nm correspond to 40 µg/ml

50 Using the value of absorbance at 280 nm calculate A_{260}/A_{280}ratio to determine purity of the sample

Citation: Jessica Gasparello, Chiara Papi, Matteo Zurlo, Lucia Carmela Cosenza, Giulia Breveglieri, Cristina Zuccato, Roberto Gambari, Alessia Finotti S2 File The Protocols of the laboratory practical lessons https://dx.doi.org/10.17504/protocols.io.b6pgrdjw

This is an open access protocol distributed under the terms of the **Creative Commons Attribution License** (https://creativecommons.org/licenses/by/4.0/), which permits unrestricted use, distribution, and reproduction in any medium,
