# Supplementary material for: Teaching during COVID-19 pandemic in practical laboratory classes of applied biochemistry and pharmacology: A validated fast and simple protocol for detection of SARS-CoV-2 Spike sequences
Source: PLoS One. 2022 Apr 6;17(4):e0266419. doi: 10.1371/journal.pone.0266419 (PMC8985952; doi:10.1371/journal.pone.0266419)

Teaching during COVID-19 pandemic in practical laboratory classes of applied biochemistry and pharmacology: a validated fast and simple protocol for detection of SARS-CoV-2 Spike sequences

Jessica Gasparello[1], Chiara Papi[1], Matteo Zurlo[1], Lucia Carmela Cosenza[1], Giulia Breveglieri[1], Cristina Zuccato[1], Roberto Gambari[1,2,*] and Alessia Finotti[1,*]

[1]Department of Life Sciences and Biotechnology, University of Ferrara, 44121 Ferrara, Italy;
[2]Interuniversity Consortium for Biotechnology (CIB), 34012 Trieste, Italy

Supporting Information S3 file

Extraction of the pCMV3-C-GFPSpark plasmid DNA

pCMV3-C-GFPSpark (a)

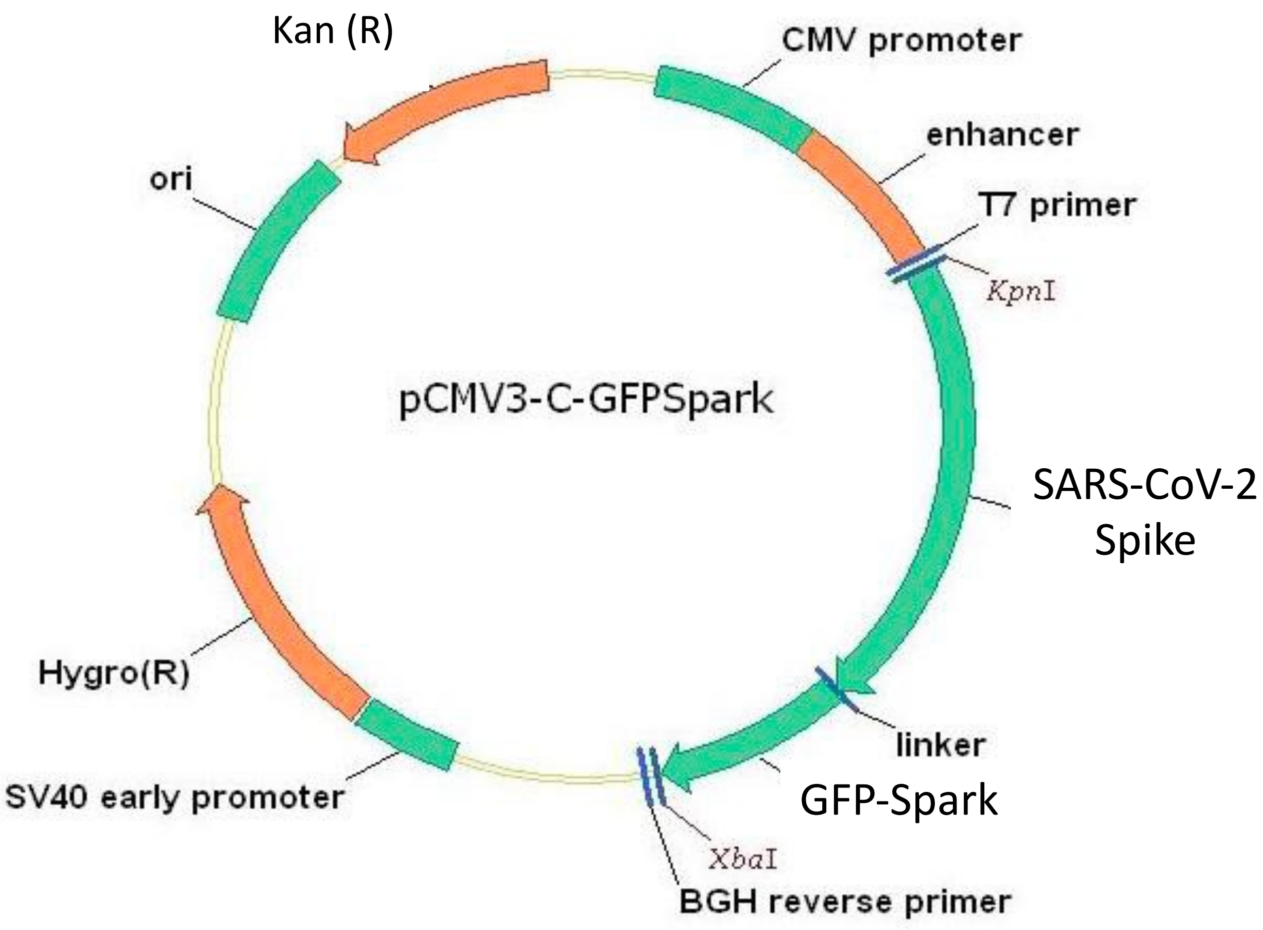

SARS-CoV-2 (2019-nCoV) Spike S1 Gene ORF cDNA clone expression plasmid, C-GFPSpark tag (Codon Optimized)

Vector size	6848 bp
Vector Type	Mammalian expression vector
Expression Method	Consitutive, Stable/Transient
Promoter	CMV
Bacterial Resistance	Kanamycin
Selection in Cells	Hygromycin
Protein tag	GFPSpark

pCMV3-Spike-GFPSpark plasmid (Cat: VG40591-ACG) was purchased by SinoBiological)

pCMV3-C-GFPSpark (b)

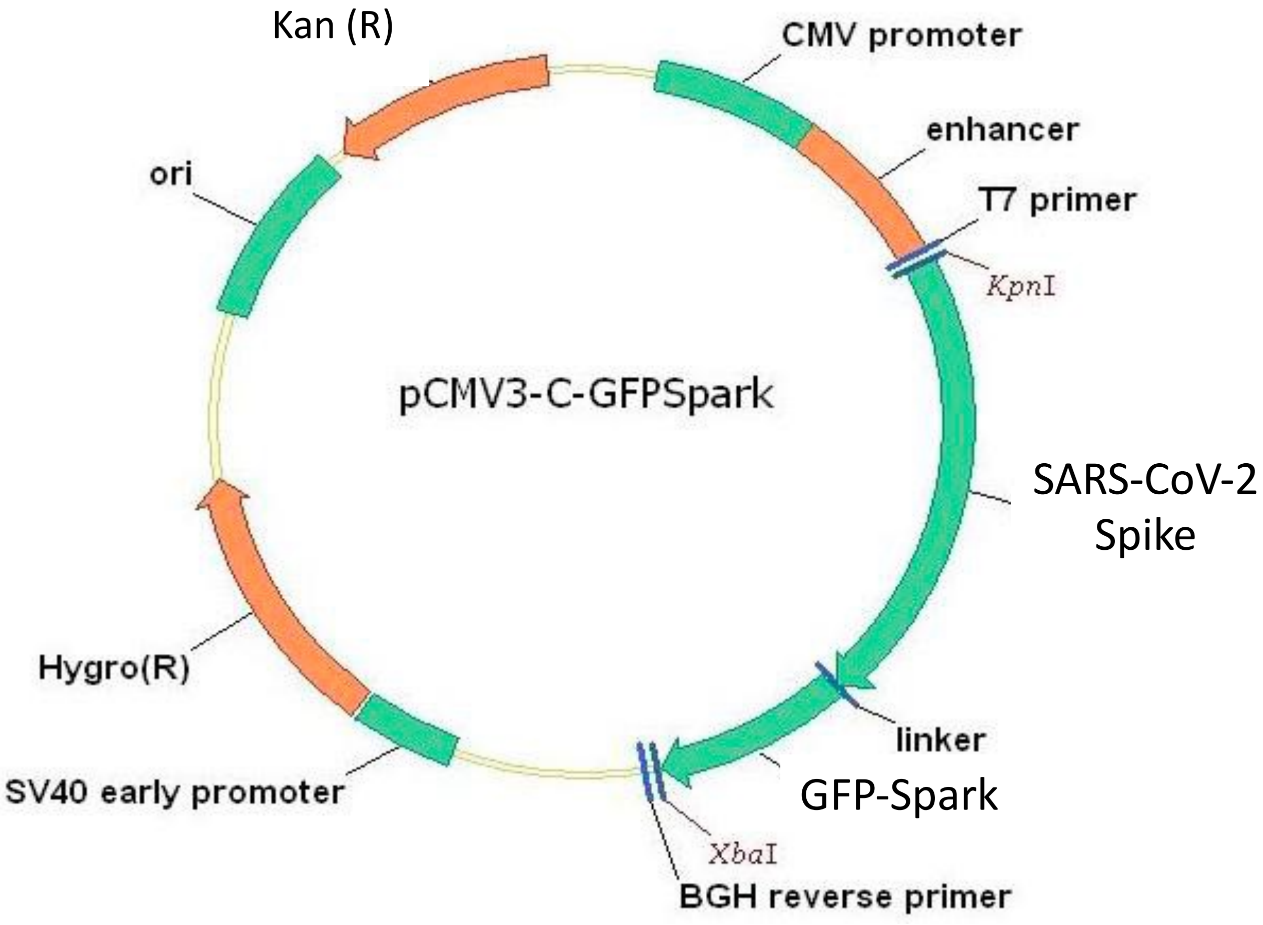

SARS-CoV-2 (2019-nCoV) Spike S1 Gene ORF cDNA clone expression plasmid, C-GFPSpark tag (Codon Optimized)

The pCMV-C-GFPSpark vector has been designed for high-level stable and transient expression in most mammalian cells.

The pCMV-C-GFPSpark vector contains the following key elements:

(a) Human enhanced cytomegalovirus immediate-early (CMV) promoter for high-level expression in a wide range of mammalian cell lines.

(b) Hygromycin resitance gene for selection of transfected mammalian cells.

(c) A Kozak consensus sequence to enhance mammalian expression.

Transformation of chemically-competent bacterial cells with pCMV-C-GFPSpark plasmid: a short description

-Thaw bacteria (*E.coli* JM109 strain, made competent by chemical treatment) on ice for about ten minutes (use about 100 µl aliquots).

-Mix bacteria by finger vortexing.

-Add 10 ng of pCMV-C-GFPSpark by slowly pipetting (no more than a couple of times) (A).

-Incubate on ice for 30 minutes (B).

-Thermal shock at 42°C for 1 minute (C).

-Put on ice for 2 minutes.

-Add 200 µl of LB (Luria-Bertani)-medium (Triptone; Yeast Extract; NaCl) without antibiotic (D).

-Incubate at 37°C for 1 hour, keeping in slow shaking (about 90 rpm) (E).

-Transfer, with a pipette, the contents of the Eppendorf in a plate (F).

-Distribute the bacteria on the plate using a loop, continue until the plate is completely dry (G).

-Then, leave the plate open under the hood for 5 minutes (H).

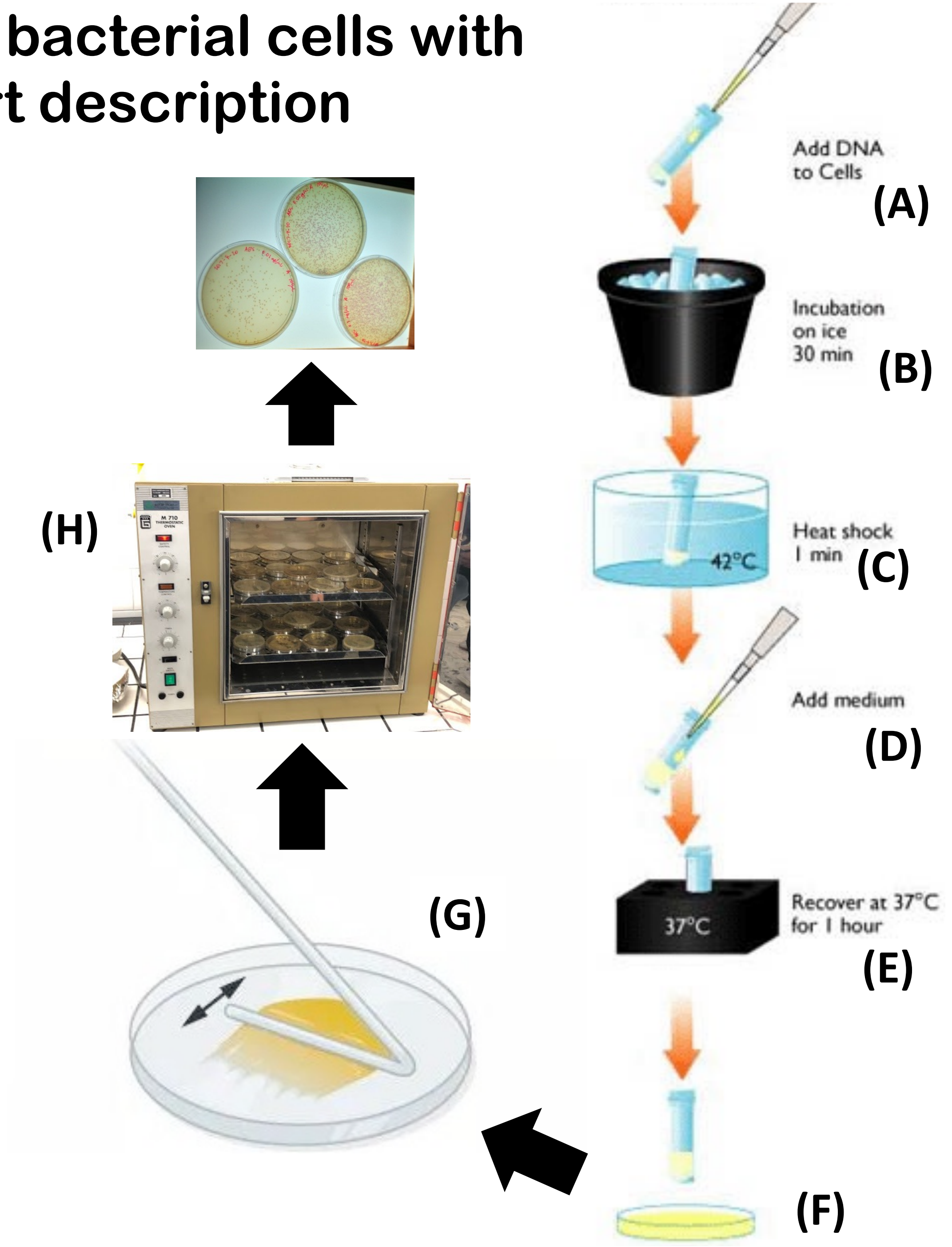

Amplification of bacterial cells carrying the pCMV-C-GFPSpark plasmid

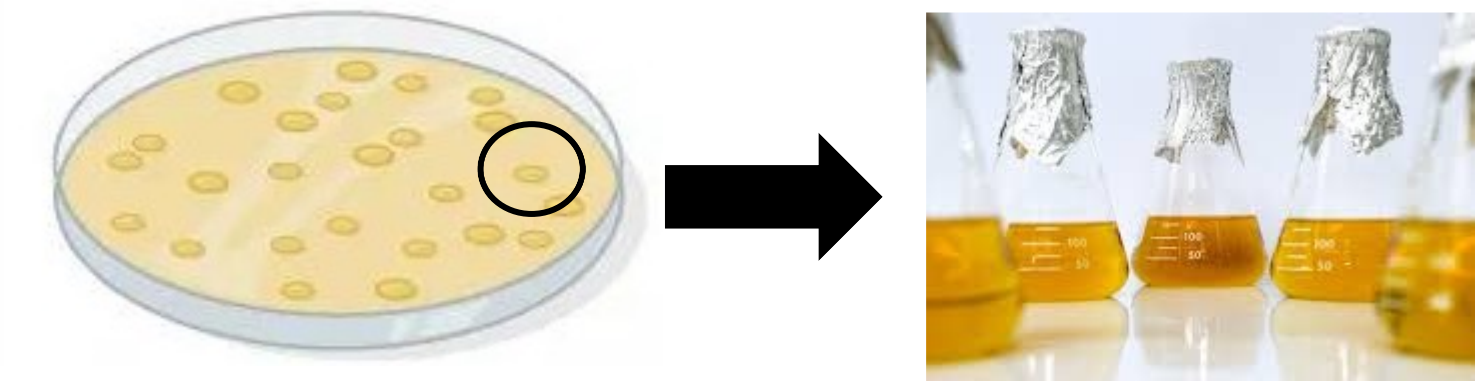

-Grow the bacteria carrying the pCMV-GFP plasmid in liquid LB-medium (Luria Bertani medium) at 37°C under shaking until the desired density is reached. Use a single colony as starting material.

Comments. (1) The bacterial cells divide approximately every 20 min, until they reach a plateau phase in which their concentration is about 10^9 cells/ml. (2) It is possible to monitor growth through the evaluation of the culture medium measuring OD (optical density) at 600 nm using a spectrophotometer. (3) The goal is to create a medium with a high number of bacterial cells that contain the plasmid inside them. These cells will be lysed to expose the plasmid which will be extracted

Isolation/extraction of the pCMV-C-GFPSpark plasmid: a short description (I)

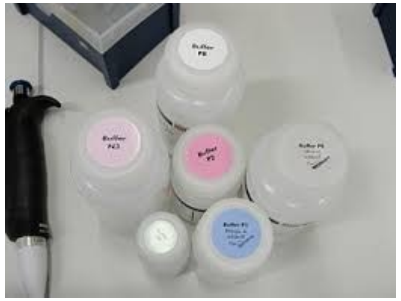

-Take about 50 ml of culture medium containing bacteria grown up to the plateau (turbid medium); centrifuge bacteria at 3000 rpm for 30 minutes at 4°C; remove the supernatant.

-Resuspend the pellet with 5 ml of buffer P1 (Qiagen, Germany): this buffer contains EDTA which binds Mg ions and inhibits nucleases (50 mM Tris·Cl, pH 8.0, 10 mM EDTA, 100 µg/ml RNase A).

-Add 4 ml of buffer P2 (Qiagen, Germany), shake by inversion for 4 times. Buffer P2 contains 200 mM NaOH and 1% SDS (w/v) (comment: white filaments should appear).

-Incubate 5 minutes room temperature (RT); add 5 ml of buffer P3/N3 (Qiagen, Germany) and shake by inversion 4 times (comment: white precipitated particles will form, composed by proteins, membrane residues and chromosomal DNA); incubate on ice for 15 minutes [comment: buffer P3/N3 contains acetic acid in percentage between 10-20% (w/w) as indicated in buffer P3/N3 safety data sheet].

-Centrifuge for 30 minutes at 4°C, 3000 rpm; take the supernatant which contais the plasmid DNA.

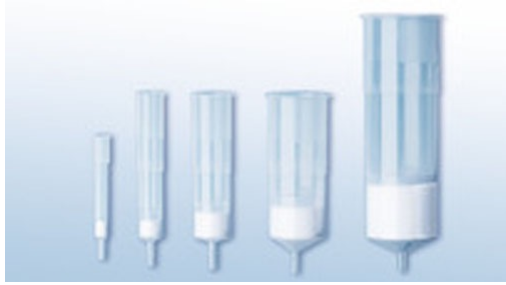

Isolation/extraction of the pCMV-C-GFPSpark plasmid: a short description (II)

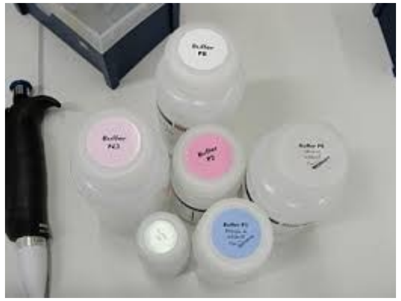

-Purify plasmid DNA through column purification.

-Elute the plasmid (comment: diluted in a large volume).

-Add 3.5 ml of isopropanol to precipitate the plasmid DNA (comment: isopropanol removes H_2O molecules from DNA precipitating DNA).

-Centrifuge at 12000 rpm for 30 minutes at 4°C.

-Discard the supernatant by inverting the tube and dry the tube using a lab paper.

-Add 400 μl of 75% EtOH to each eppendorf [comment: a white 'feather' (precipitated plasmid DNA) is formed (EtOH solution is used to eliminate salts from DNA)].

-Centrifuge at 12000 rpm 4°C for 10 minutes.

-Remove the ethanol with a syringe.

-Allow the pellet to dry in the air, under the wood; resuspend plasmid DNA pellet in TE (Tris EDTA pH = 8) (**ready for the laboratory classes**).

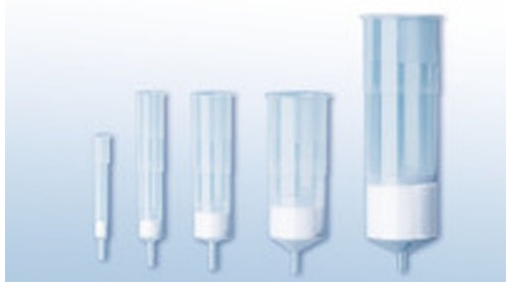

Comments to Plasmid DNA extraction

(1) Bacterial cells contain both chromosomal and plasmid DNA, to isolate only the plasmid DNA, one of the most used techniques is ALKALINE LYSIS.

(2) Alkaline lysis is based on the use of a NaOH-based solution, which, added to the bacterial lysate, allows to reach pH values of about 12-12.5. At this pH value, DNA denatures because the hydrogen bonds that hold together the double-stranded structure are broken. When the pH is brought back to physiological values by adding acid solutions (e.g. potassium acetate): Chromosomal DNA aggregates to form an insoluble precipitate, which can be eliminated by centrifugation. Plasmid DNA reassembles in the super-coiled conformation and remains in solution.

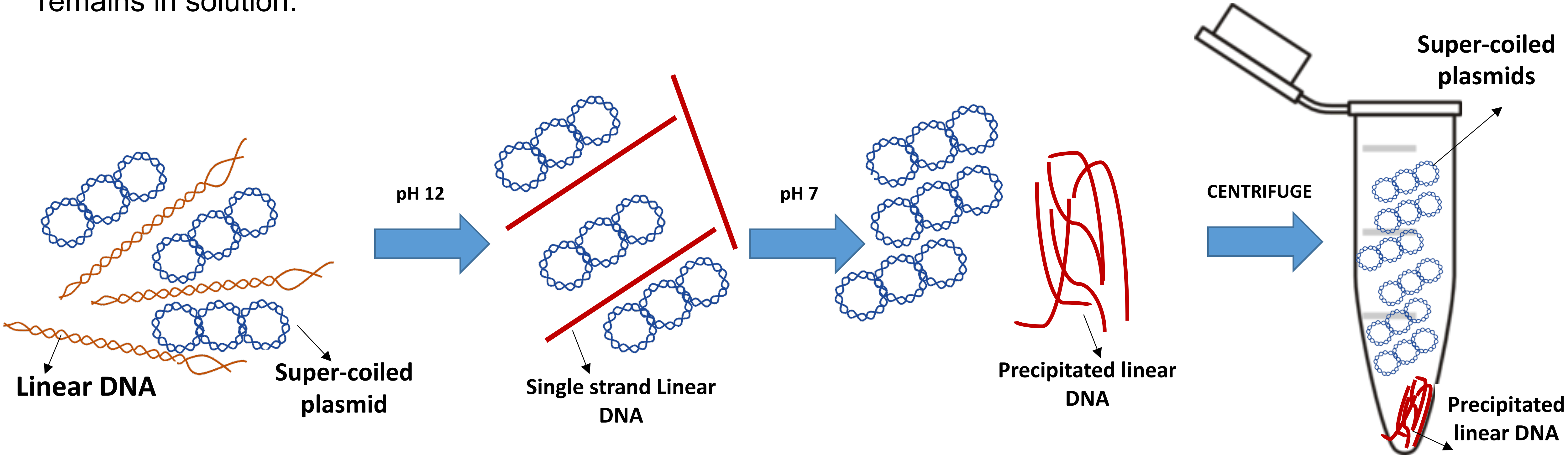

Supplement: S3 File — (PDF) [file pone.0266419.s003.pdf]
