# Supplementary material for: Teaching during COVID-19 pandemic in practical laboratory classes of applied biochemistry and pharmacology: A validated fast and simple protocol for detection of SARS-CoV-2 Spike sequences
Source: PLoS One. 2022 Apr 6;17(4):e0266419. doi: 10.1371/journal.pone.0266419 (PMC8985952; doi:10.1371/journal.pone.0266419)
Supplement: S4 File — (PDF) [file pone.0266419.s004.pdf]

Teaching during COVID-19 pandemic in practical laboratory classes of applied biochemistry and pharmacology: a validated fast and simple protocol for detection of SARS-CoV-2 Spike sequences

Jessica Gasparello[1], Chiara Papi[1], Matteo Zurlo[1], Lucia Carmela Cosenza[1], Giulia Breveglieri[1], Cristina Zuccato[1], Roberto Gambari[1,2,*] and Alessia Finotti[1,*]

[1]Department of Life Sciences and Biotechnology, University of Ferrara, 44121 Ferrara, Italy;
[2]Interuniversity Consortium for Biotechnology (CIB), 34012 Trieste, Italy

Supporting Information S4 file

Transfection of A549 cells with pCMV3-C-GFPSpark plasmid

A549

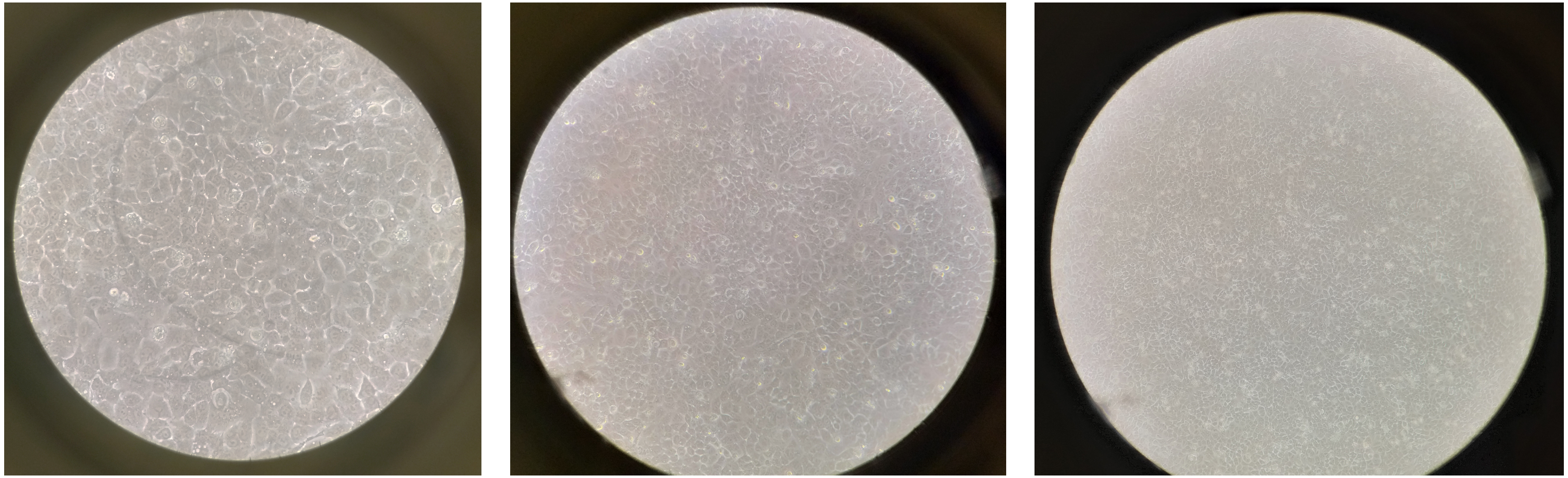

Comment: This line was developed in 1972 by D.J. Giard, et al. through explant culturing of lung carcer tissue from a 58-year-old Caucasian male. A549 cells have served as models of alveolar Type II pulmonary epithelium, finding utility in research studies examining the metabolic processing of lung tissue and possible mechanisms of drug delivery. In the context of lung cancer drug development, the cells have served as testing novel drugs (such as paclitaxel, docetaxel, and bevacizumab) both *in vitro* and *in vivo*. A549 has also been employed in viral research and associated protein expression changes as a consequence of viral infection.

Cell transfection: introductory remarks (I)

TRANSFECTION: transfer exogenous DNA into mammalian cells, in our case A549 cells.

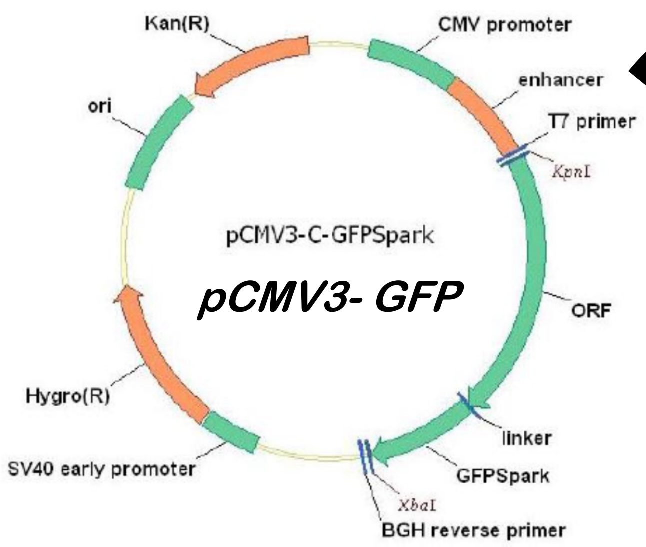

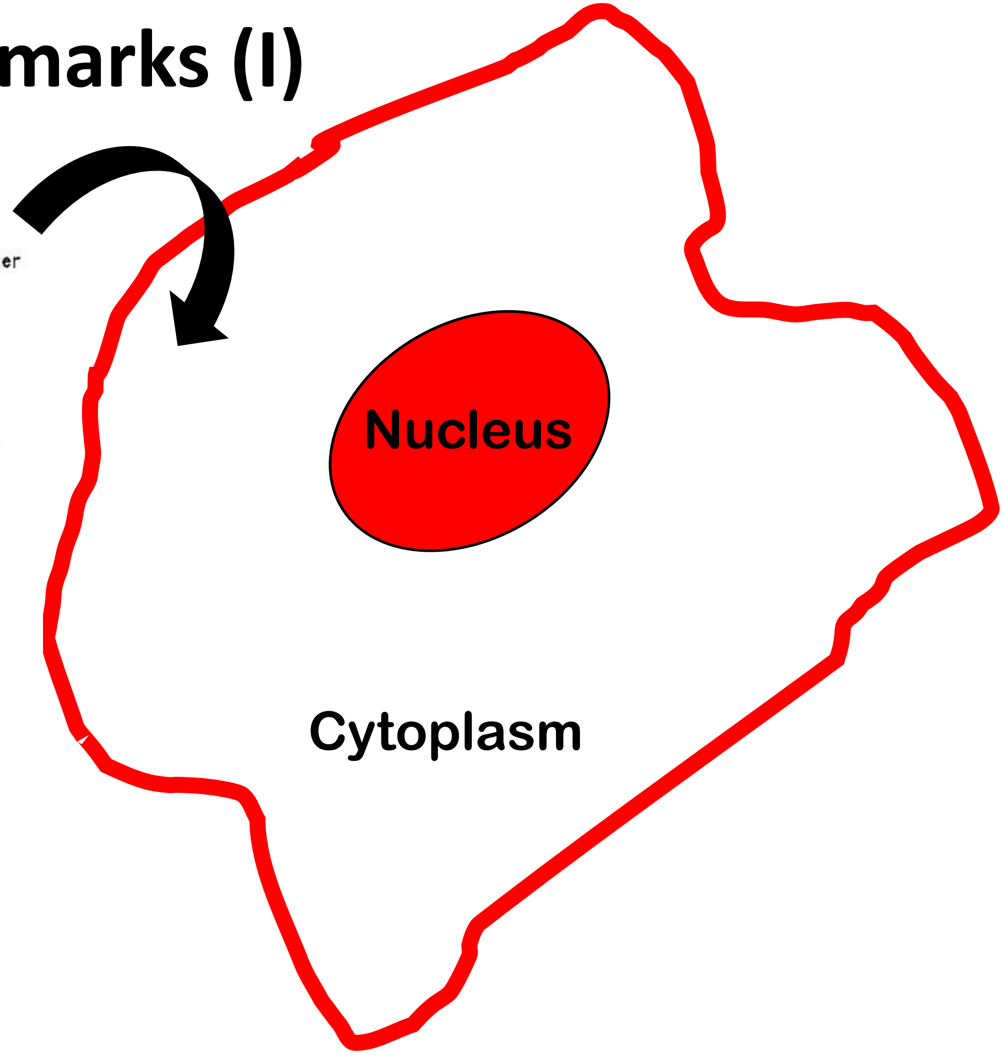

Transfection features
- ✓ High efficiency
- ✓ Low toxicity
- ✓ In vitro and in vivo reproducibility

Transfection methods:

Transient

- Transfected DNA does not duplicate, for this reason at each mitosis, cells double and the amount of DNA transfected in relation to the cells is halved
- It is used for short-term experiments: cells are harvested 48-72 h after transfection
- NOT homogeneous cell population
- Rapid

Stable

- For long-term experiments
- Possibility of isolating and propagating single clones
- The plasmid integrates into the host cell genome but integration is random
- Selection marker needed (e.g. antibiotic)
- Homogeneous cell population

Cell transfection: introductory remarks (II)

Liposomes for cellular transfection

- Mixture of polycationic and neutral lipids, allows the formation of unilamellar liposomal vesicles with positive charge
- The positive charge of the liposome interacts with the phosphate groups (negative charge) of the nucleic acid
- Lipid-DNA complexes fuse with cell membranes, enter the cell by endocytosis and release their contents into the cells

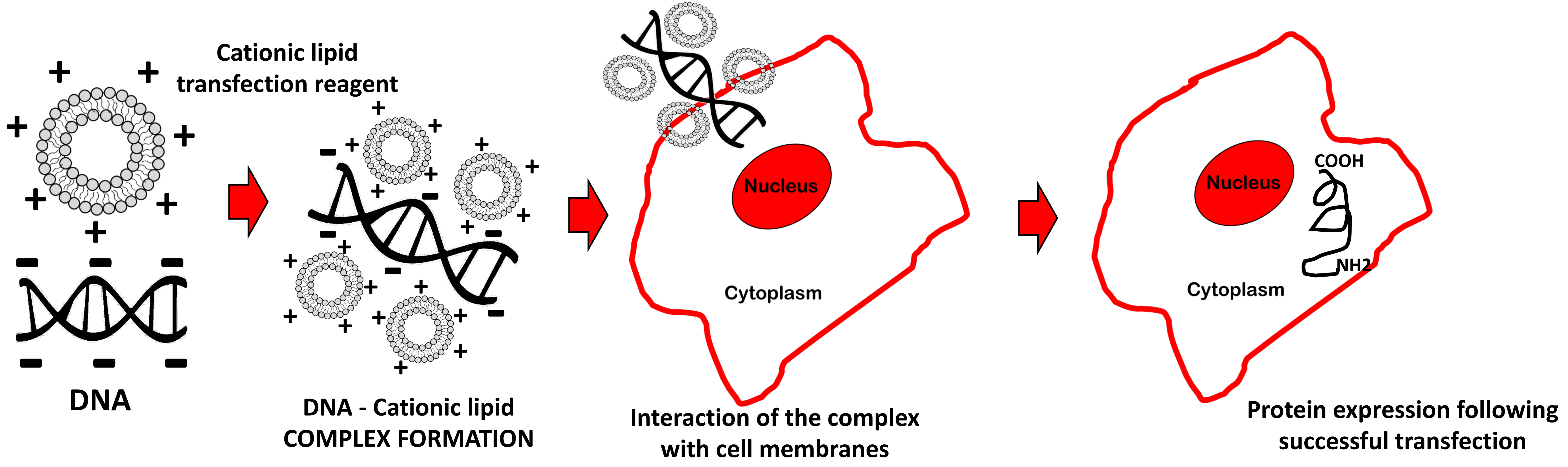

Cell transfection: introductory remarks (III)

Advantages and possible drawbacks of liposomes for cellular transfection

Advantages

- Possibility of transfecting a wide range of cell types
- Possibility of carrying both DNA (wide range of sizes) and RNA
- Can be obtained both transient and stable transfections
- Can be used both in vitro and in vivo (animal + human models)

Disadvantages

Transfection efficiency depends on
- ✓ cell line
- ✓ culture conditions (e.g. presence of serum)

Transcription of A549 cells with pCMV3-C-GFPSpark plasmid: a short description

-24 hours before transfection, plate A549 cells in 12 wells plate. Cells must be 70% confluent at the day of transfection.

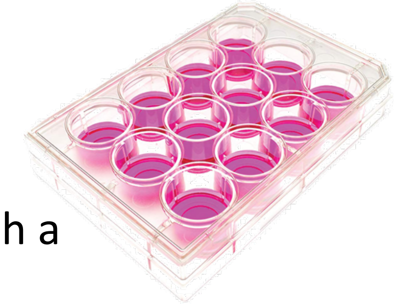

-At day of transfection replace medium with a new one, 30 min before the transfection procedure.

-Prepare a mix containing 1 µg of plasmid DNA, 1 µl of Plus Reagent and Opti-MEM/Reduced Serum, reaching the final volume of 47.5 µl. Incubate the mix for 5 min RT (room temperature).

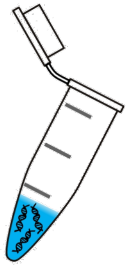

-Add to the mixture 2.5 µl of Lipofectamine LTX and incubate for 30 min at RT.

-After the incubation add the transfection mixture to the cells and maintain the mixture in contact with the cells for 24 h, then replace with fresh medium.

-24 hours after the transfection cells can be visualized using a fluorescence microscope or analyze by FACS analysis

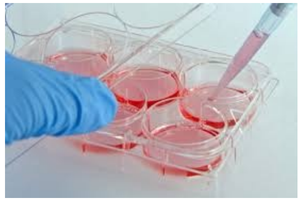

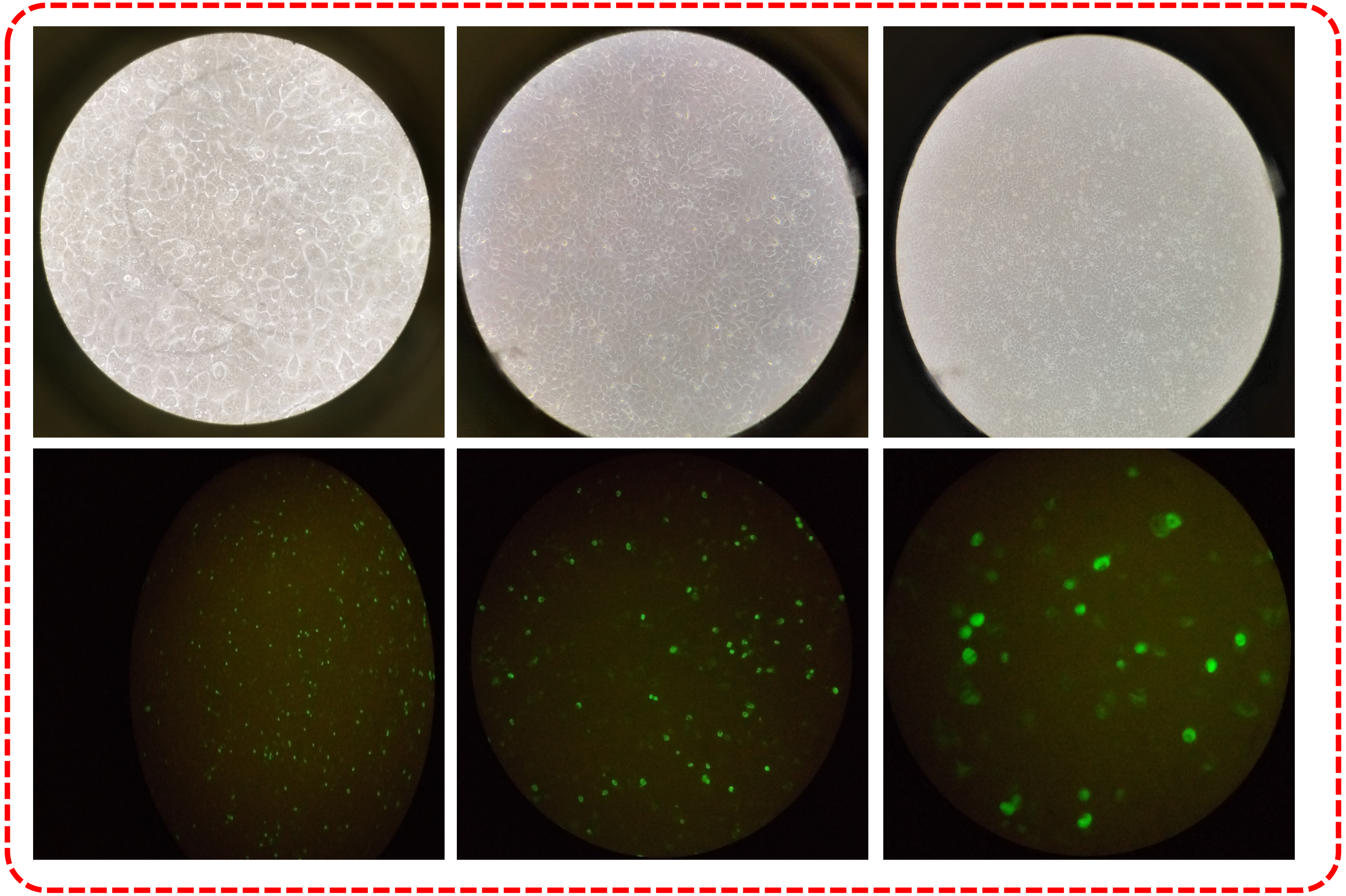

GFP: introductory remarks

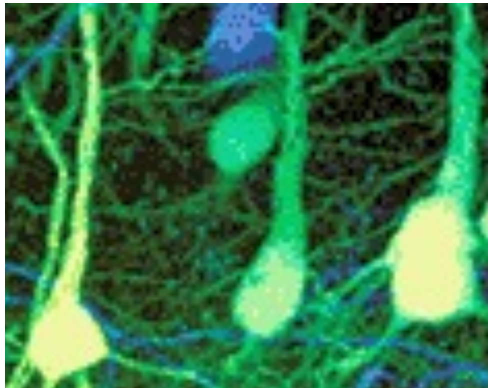

Green fluorescent protein (GFP) is a 238 amino acid protein with a molecular weight of 27 KDa identified in 1962. GFP was isolated from Aequorea Victoria jellyfish. GFP is excited at a wavelength of 488 nm and has an emission peak at 510 nm. Scientist manipulate the protein structure and engineer color variants that emit at different wavelengths.

Applications of GFP-based approaches.
(1) To study the localization of proteins in cellular compartments.
(2) To study molecular pathways: interactions between proteins lead to variations in fluorescence (FRET).
(3) As cellular sensors (e.g. fluorescence variations in relation to the pH values of a certain cell compartment).

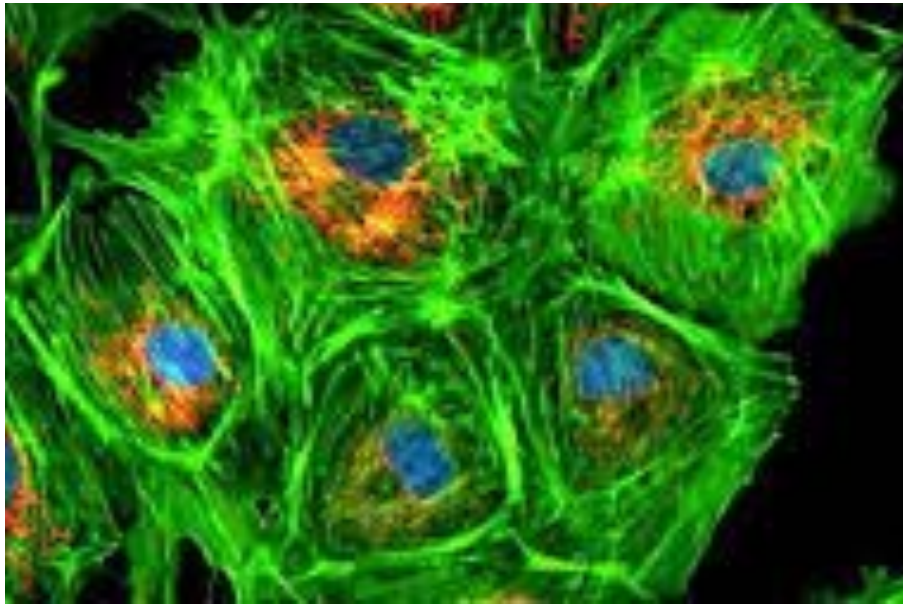

The importance of GFP was recognized in 2008 when the Nobel Committee awarded Osamu Shimomura, Marty Chalfie and Roger Tsien the Chemistry Nobel Prize "for the discovery and development of the green fluorescent protein, GFP."

Flow Cytometry-FACS: introductory remarks

Flow cytometry

- used for the analysis of cells in suspension that are in a laminar flow, which allows to characterize the cells from a morphological and biochemical point of view.
  - FACS: Fluorescence-activated Cell Sorting (registered trademark).
    - Sorting = separates cells based on their features.

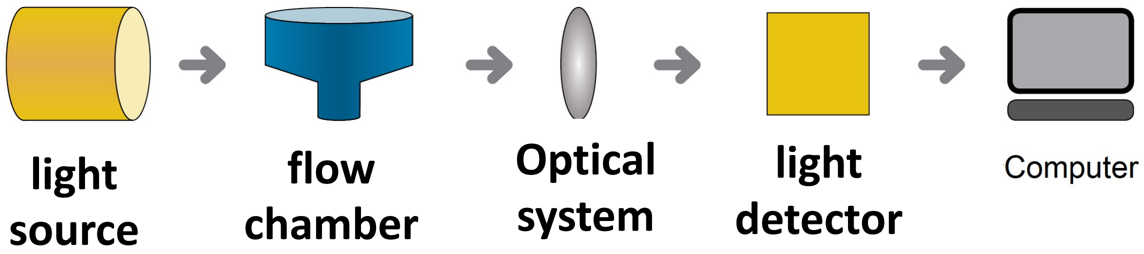

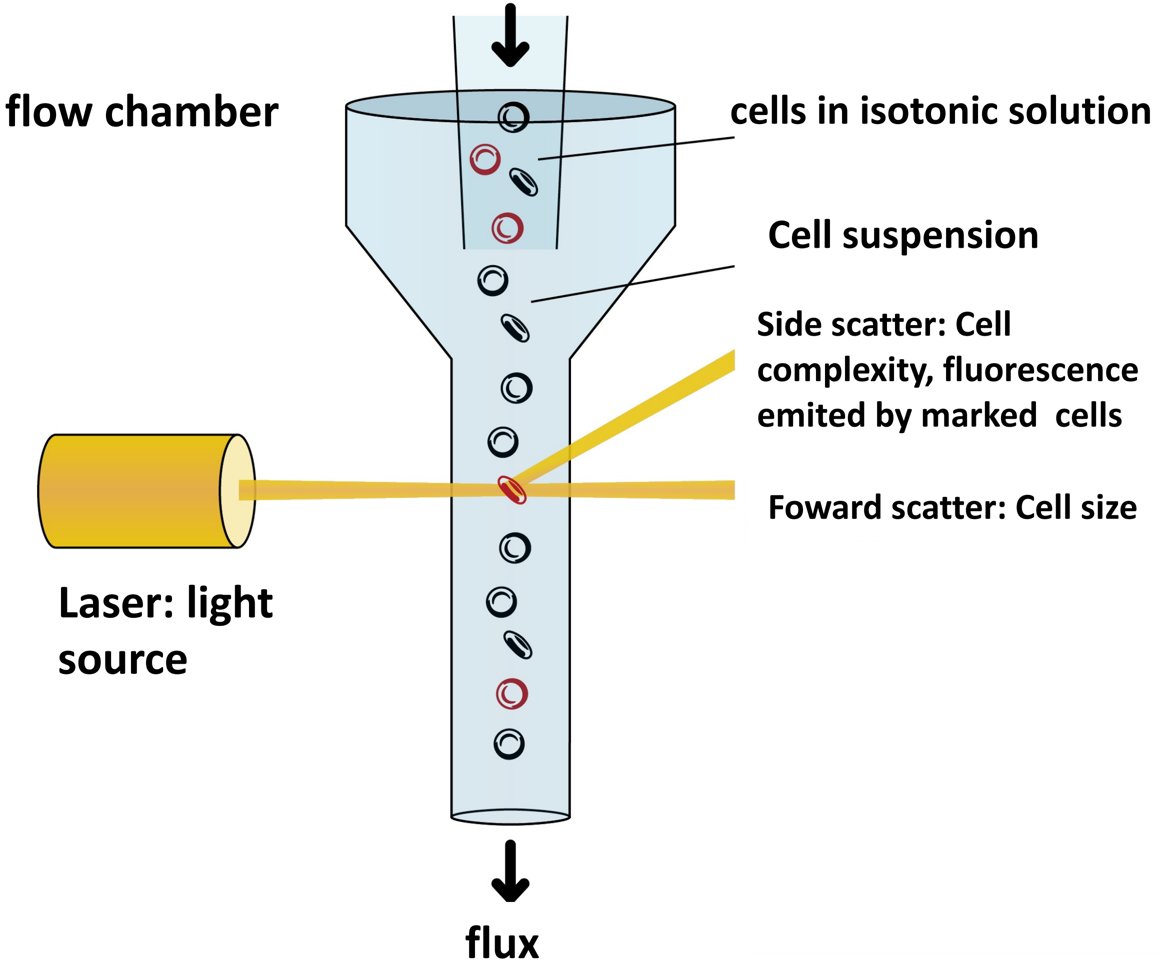

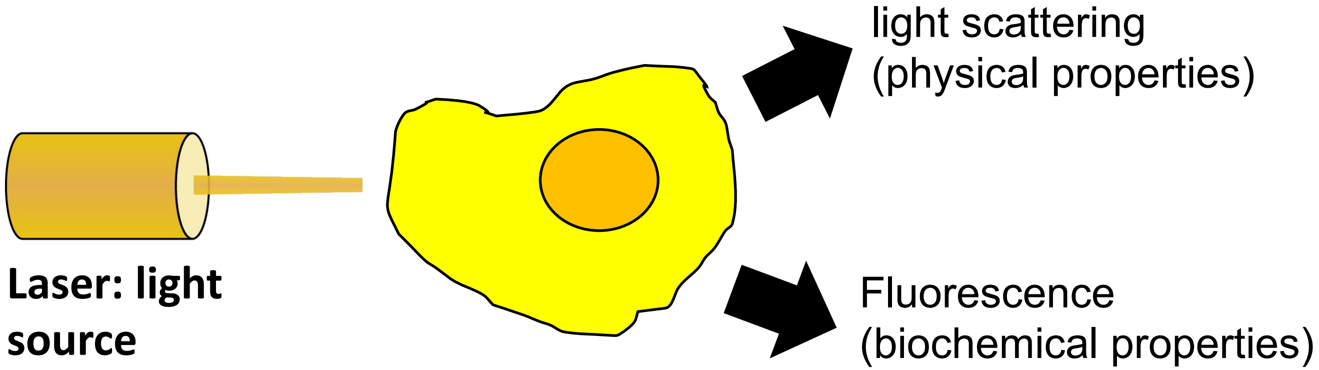

FACS: representative histograms

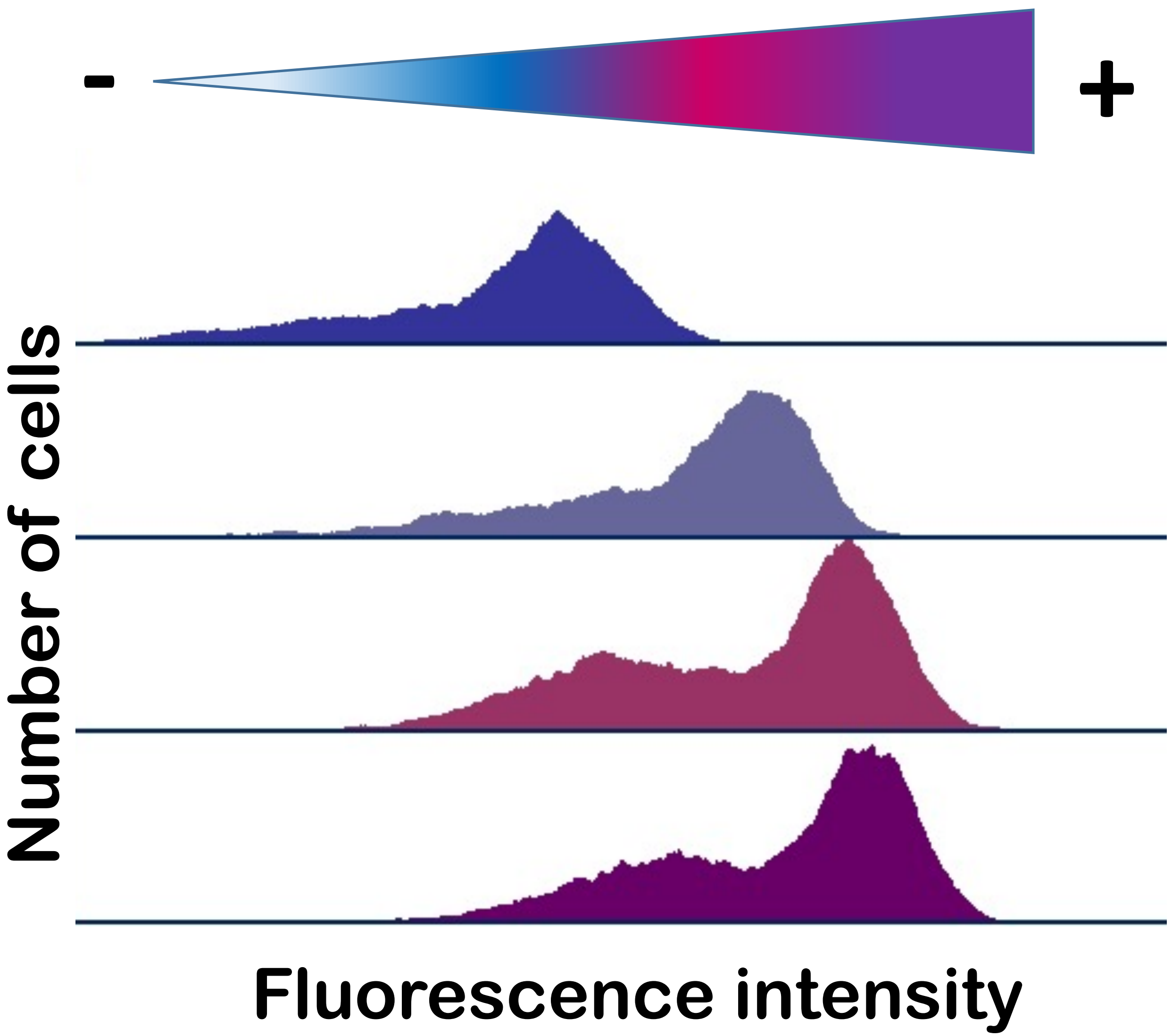

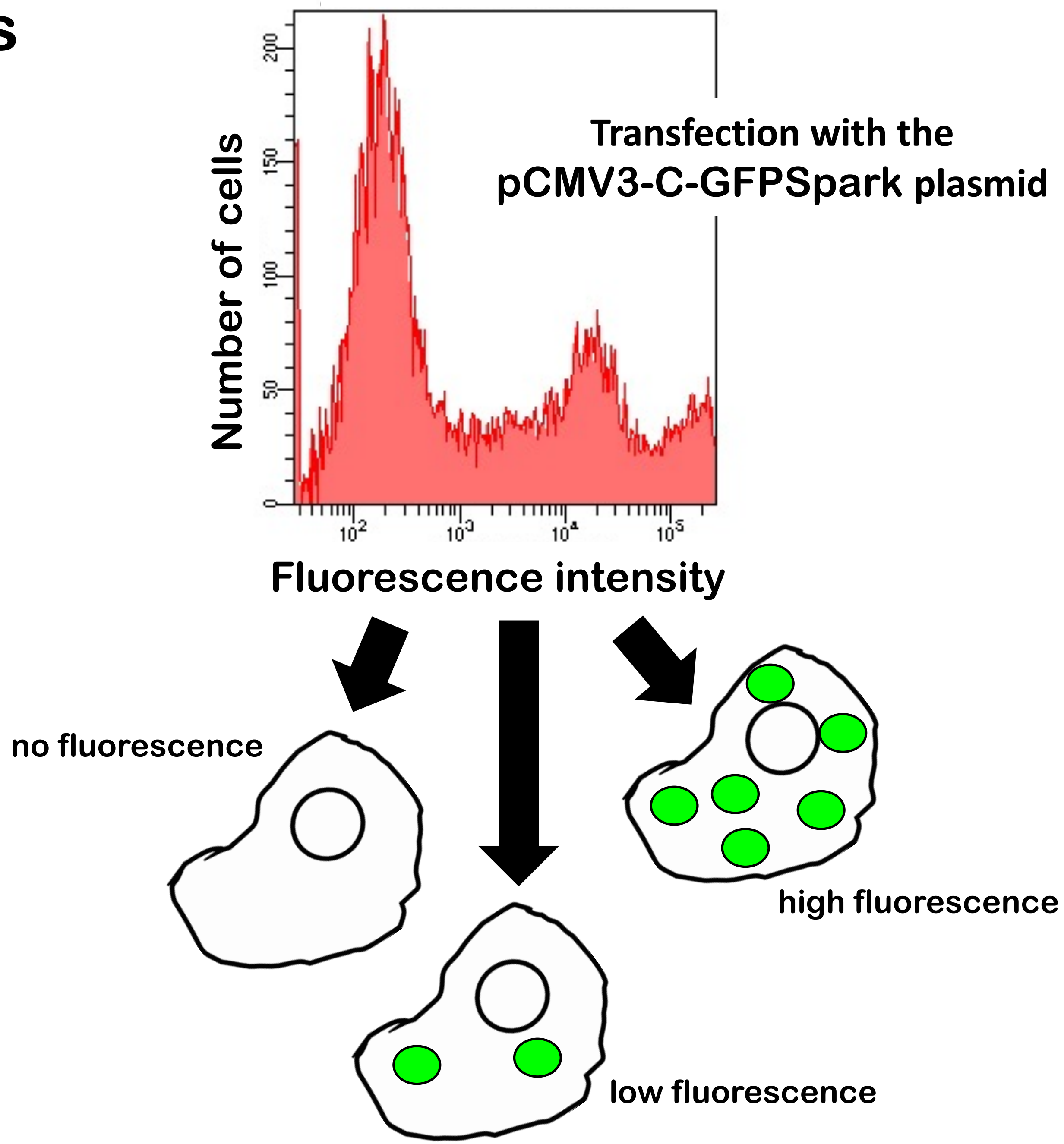

FACS-based characerization of A549 cells transfected with pCMV3-C-GFPSpark plasmid: key results

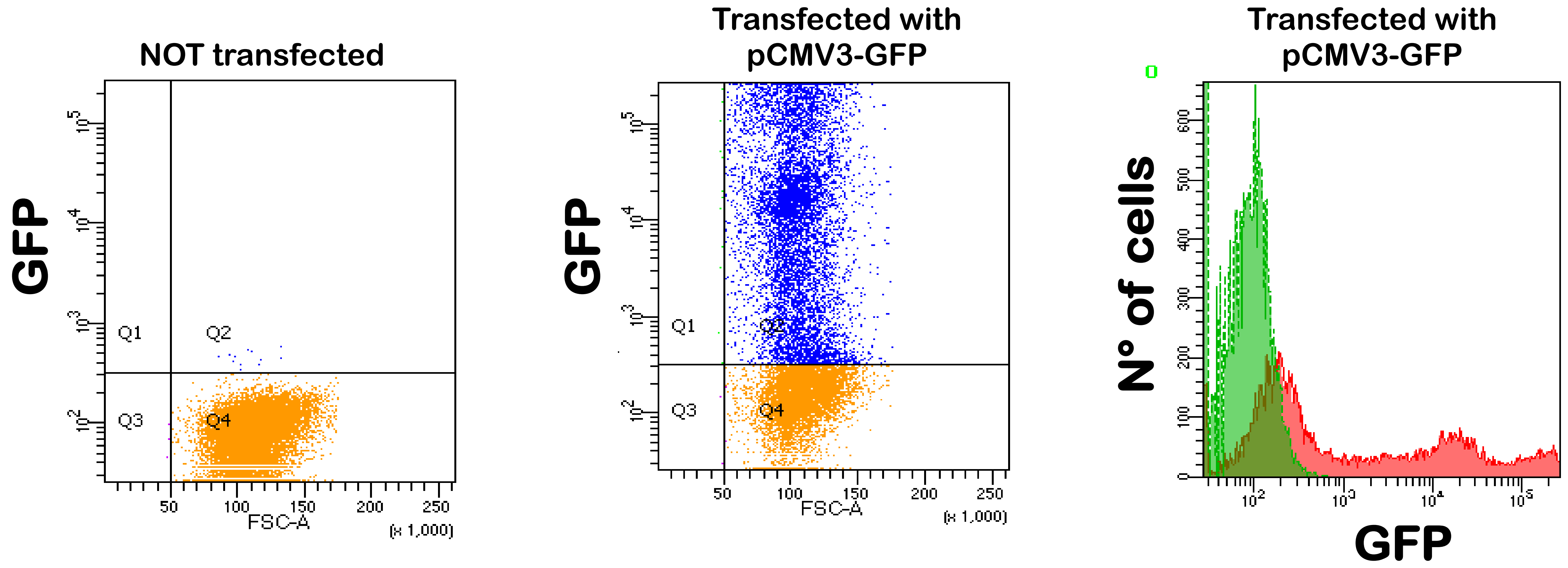

The A549 cells characterized for transfection with the pCMV-C-GFPSpark plasmid are ready for RNA extraction for the laboratory classes.

FACS analysis: a short description

-Remove the supernatant from the cell cultures, and detach the cells by incubation for 5 min with a trypsin-EDTA solution, at RT (room temperature).

-Replace the starting culture volume with fresh medium.

-Centrifuge at 1200 rpm for 10 min at RT.

-Remove supernatant.

-Resuspend cell pellets with 300 µl of Dulbecco's phosphate-buffered saline solution (DPBS) with 1% FBS. Transfer suspended cells in a FACS-Tube.

-Analyze green fluorescence (FITCH channel) of the cells with a FACS instrument, acquiring at least 30000 events
