# Supplementary material for: Teaching during COVID-19 pandemic in practical laboratory classes of applied biochemistry and pharmacology: A validated fast and simple protocol for detection of SARS-CoV-2 Spike sequences
Source: PLoS One. 2022 Apr 6;17(4):e0266419. doi: 10.1371/journal.pone.0266419 (PMC8985952; doi:10.1371/journal.pone.0266419)
Supplement: S5 File — (PDF) [file pone.0266419.s005.pdf]

Teaching during COVID-19 pandemic in practical laboratory classes of applied biochemistry and pharmacology: a validated fast and simple protocol for detection of SARS-CoV-2 Spike sequences

Jessica Gasparello[1], Chiara Papi[1], Matteo Zurlo[1], Lucia Carmela Cosenza[1], Giulia Breveglieri[1], Cristina Zuccato[1], Roberto Gambari[1,2,*] and Alessia Finotti[1,*]

[1]Department of Life Sciences and Biotechnology, University of Ferrara, 44121 Ferrara, Italy;
[2]Interuniversity Consortium for Biotechnology (CIB), 34012 Trieste, Italy

Supporting Information S5 file

RNA extraction

RNA extraction with Phenol/Chloroform: introductory scheme

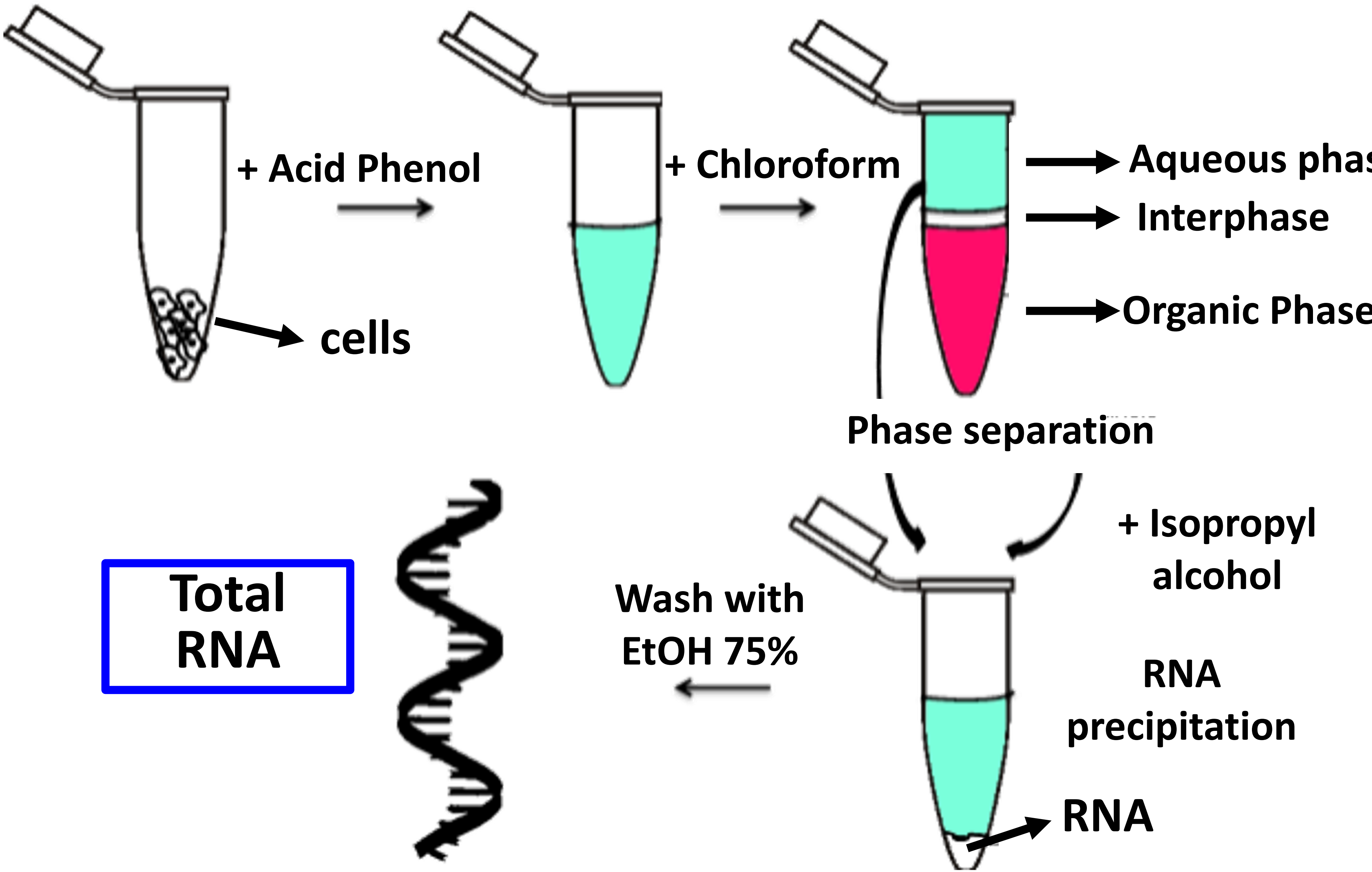

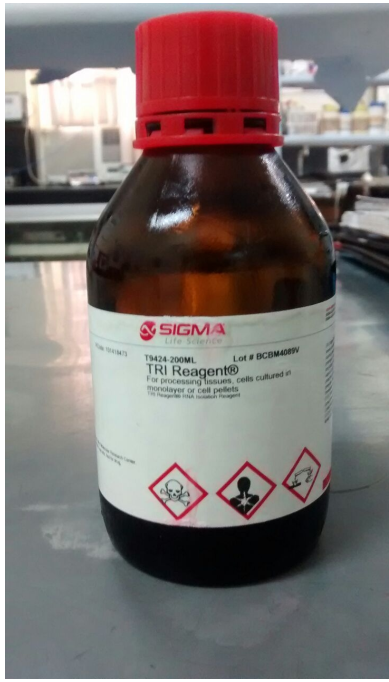

Protocol: a short description (a)

-Lyse each cellular pellet adding 900 µl of TRI Reagent using ‘p1000’ pipette, under a chemical hood: immediately after addition, pipette quickly to make the system homogeneous (no white fragments of the pellet should be present).

-Incubate the sample for 5 minutes at room temperature (RT) to dissociate the nucleoproteins from the nucleic acids, keeping it in the rack.

-Add, using the p1000, 200 µl of RNase-free chloroform, under a chemical hood. Chloroform is very volatile and tends to drip, to avoid this, prime the tip before taking the chloroform. Read MSDS (Material Safety Data Sheet).

-Mix vigorously by inversion for 15 sec, until the solution becomes homogeneous and the two phases can no longer be distinguished.

-Incubate the sample at RT for 2-3 minutes, keeping it in the rack.

RNA Extraction: introductory remarks (I)

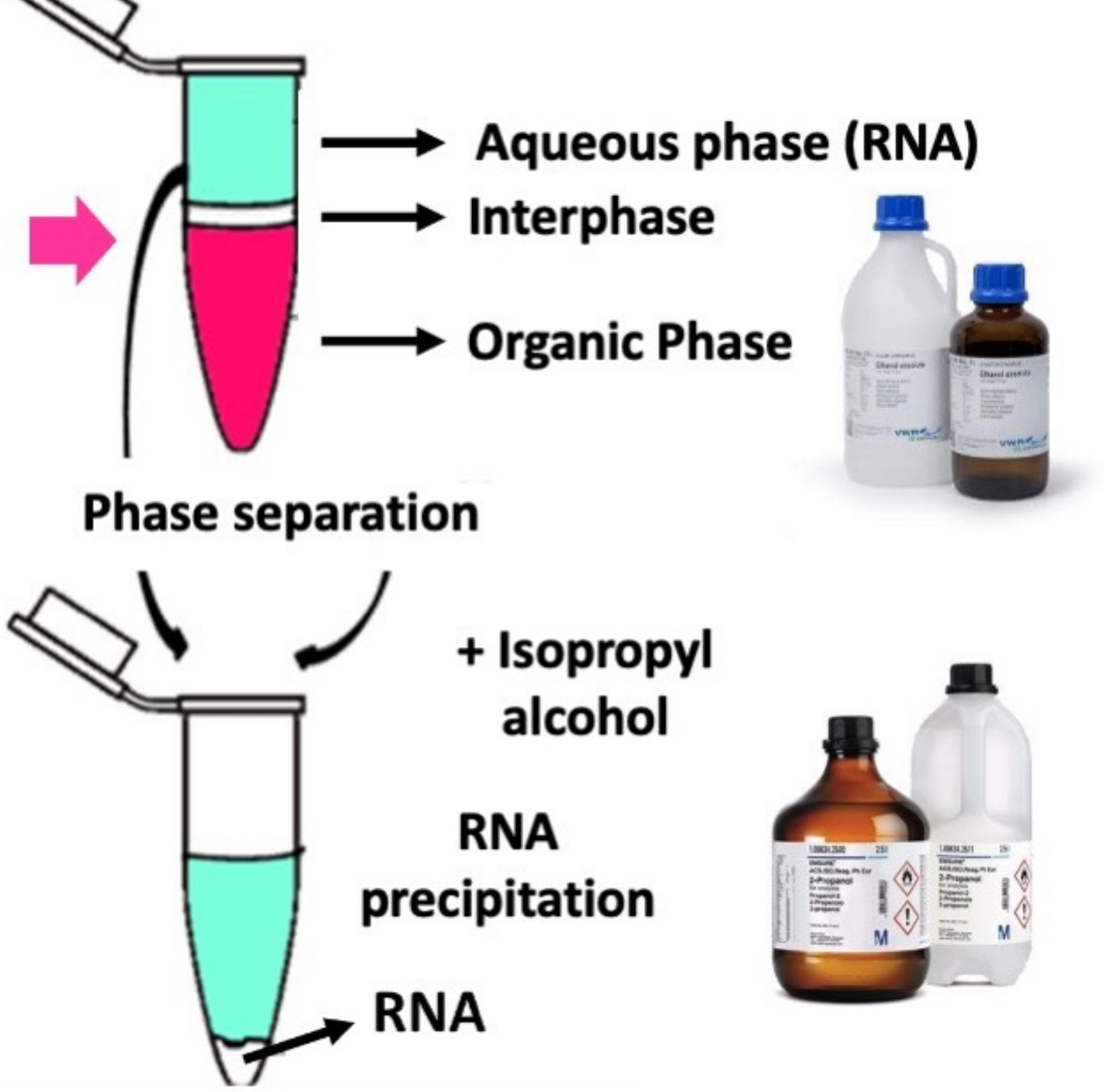

Comment: the addition of chloroform causes phase separation. Three phases can be generated:

- Organic phase (pink) contains proteins and lipids,
- Interphase (white) contains DNA
- Aqueous phase (transparent) contains RNA

Protocol: a short description (b)

-Centrifuge at 12000 rpm, for 15 minutes, at 4 ° C. The mixture separates into 3 phases:

1. Upper aqueous phase: transparent, contains RNA.
2. Whitish semi-solid interface: contains DNA and proteins.
3. Lower organic phase: pink, contains DNA, proteins and lipids.

-Take the upper aqueous phase with p200, without touching or drawing the interface or the pink organic phase, and transfer it into a new 1.5 ml RNase-free tube: work under a chemical hood. Read MSDS (Material Safety Data Sheet).

-Add 500 µl of 100% RNase-free isopropanol to precipitate the RNA and mix well by inverting the tube. As chloroform, also isopropanol is very volatile and can drip, to avoid this, prime the tip before taking the chloroform.

-Incubate the sample at RT for 10 minutes.

RNA Extraction: introductory remarks (II)

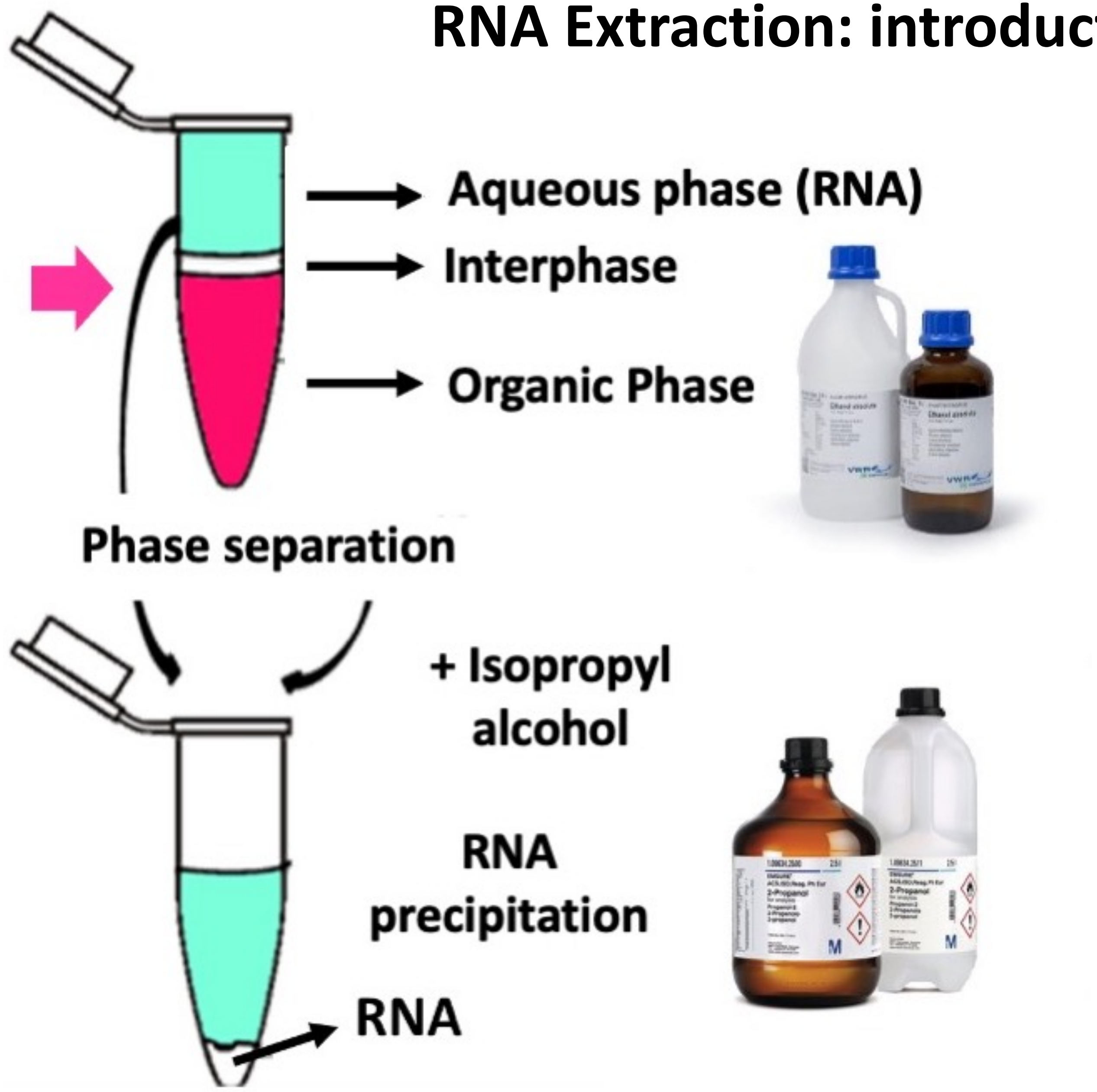

Comment: the addition of isopropanol to the aqueous phase causes RNA precipitation. Isopropanol removes the hydration shell of H_2O molecules around the phosphate groups, causing precipitation of RNA. A small pellet is formed at the bottom of the tube.

RNA Extraction: final remarks

Total RNA

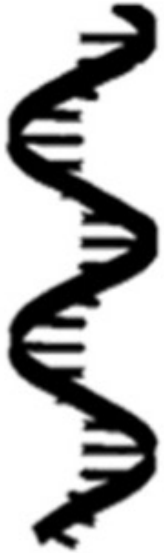

Wash with EtOH 75%

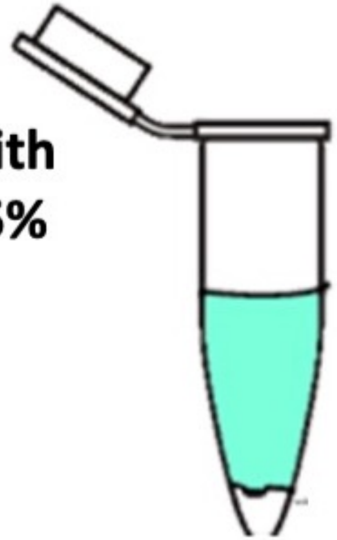

Comment: RNA wash with a solution of ethanol and water (75% ethanol, 25% water) eliminates residual salts that could contaminate the RNA. Ethanol is then removed completely to resuspend the pellet in water.

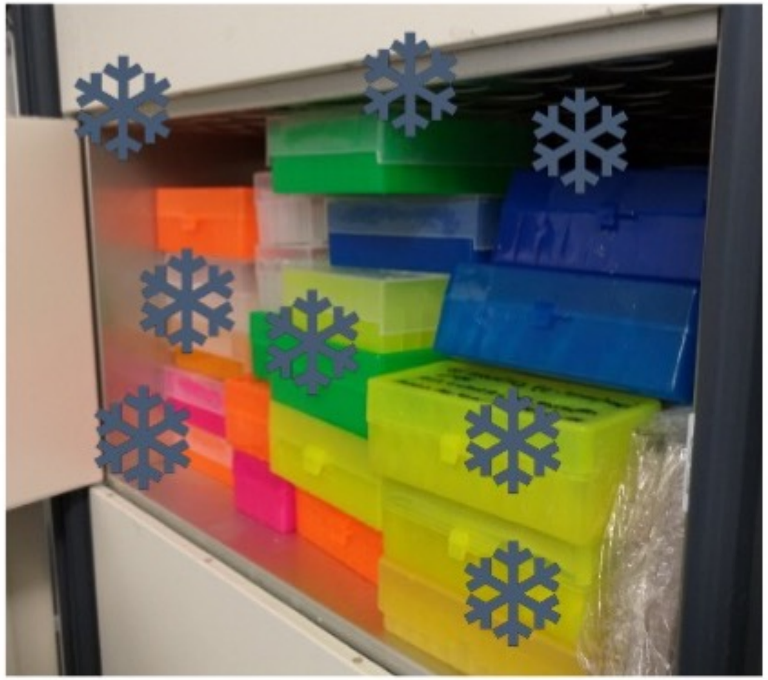

Store samples at -80°C

Protocol: a short description (c)

-Centrifuge at 12000 rpm, for 15 minutes, at 4 ° C. A pellet containing the RNA will be obtained.

-Discard the supernatant with the pipette.

-Add 900 µl of 75% RNase-free ethanol, pre-cooled on ice, the ethanol is very volatile and can drip, to avoid this, prime the tip before taking the chloroform.

-Gently invert the tube to wash the pellet. Observe carefully the size of the pellet obtained, this is essential to establish the volume of water to be used to resuspend the pellet.

-Centrifuge at 12000 rpm, for 5 minutes, at 4 ° C.

-Discard the supernatant with the pipette.

-Allow the RNA pellet to dry in the air under the hood for about 10 minutes.

-Resuspend the RNA in 20 µl or more of RNase-free H_2O, pipetting several times (the volume of water to be added may vary based on the size of the pellet and will be established after careful observation of the pellet).

-Store on ice, if analyzed immediately, or freeze at -80 ° C (**ready for the laboratory classes**).
