# Supplementary material for: Teaching during COVID-19 pandemic in practical laboratory classes of applied biochemistry and pharmacology: A validated fast and simple protocol for detection of SARS-CoV-2 Spike sequences
Source: PLoS One. 2022 Apr 6;17(4):e0266419. doi: 10.1371/journal.pone.0266419 (PMC8985952; doi:10.1371/journal.pone.0266419)
Supplement: S6 File — (PDF) [file pone.0266419.s006.pdf]

Teaching during COVID-19 pandemic in practical laboratory classes of applied biochemistry and pharmacology: a validated fast and simple protocol for detection of SARS-CoV-2 Spike sequences

Jessica Gasparello[1], Chiara Papi[1], Matteo Zurlo[1], Lucia Carmela Cosenza[1],

Giulia Breveglieri[1], Cristina Zuccato[1], Roberto Gambari[1,2,*] and Alessia Finotti[1,*]

[1]Department of Life Sciences and Biotechnology, University of Ferrara, 44121 Ferrara, Italy;
[2]Interuniversity Consortium for Biotechnology (CIB), 34012 Trieste, Italy

**Correspondence to:*

Professor Roberto Gambari, Department of Life Sciences and Biotechnology, University of Ferrara, Via Fossato di Mortara n.74, 44121 Ferrara, Italy; Tel: +39-0532-974443; Fax: +39-532-974500; email: gam@unife.it

Professor Alessia Finotti, Department of Life Sciences and Biotechnology, University of Ferrara, Via Fossato di Mortara n.74, 44121 Ferrara, Italy; Tel: +39-0532-974510; Fax: +39-532-974500; email: alessia.finotti@unife.it

Supporting Information
S6_raw_images

1. Raw uncropped images used to prepare Figure 3

1a. Figure 3

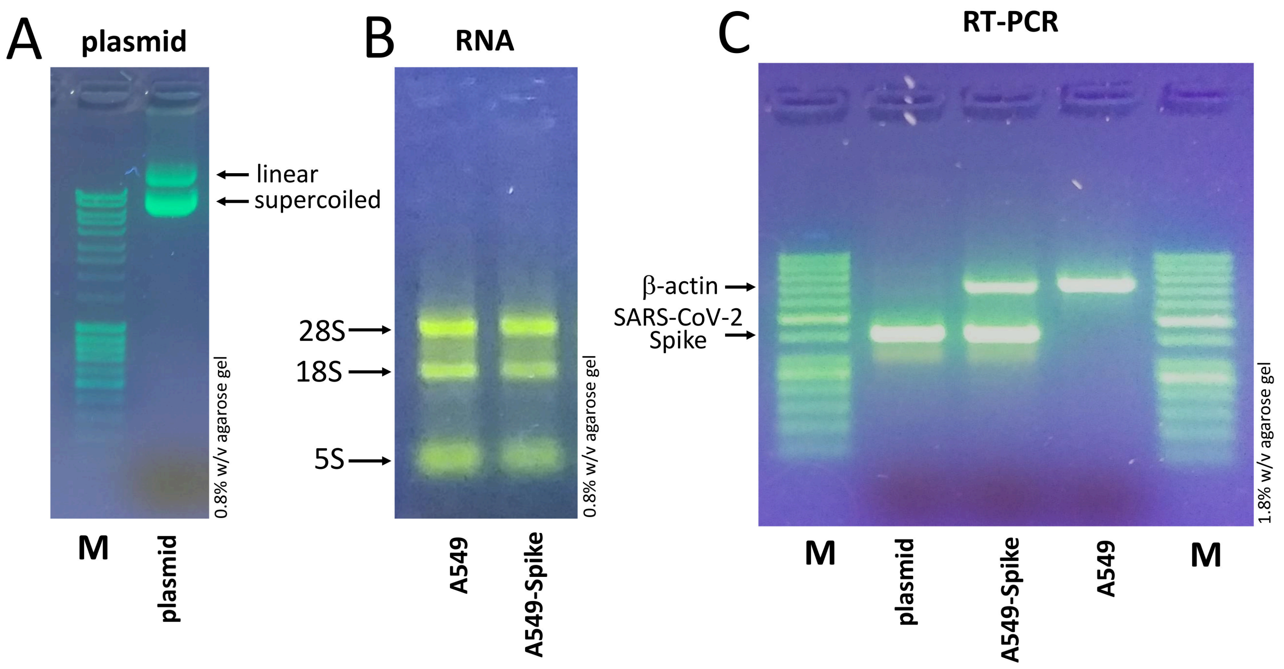

1b. Raw data used for Figure 3A

(the first two lanes were used; X = lanes not used in the Figure)

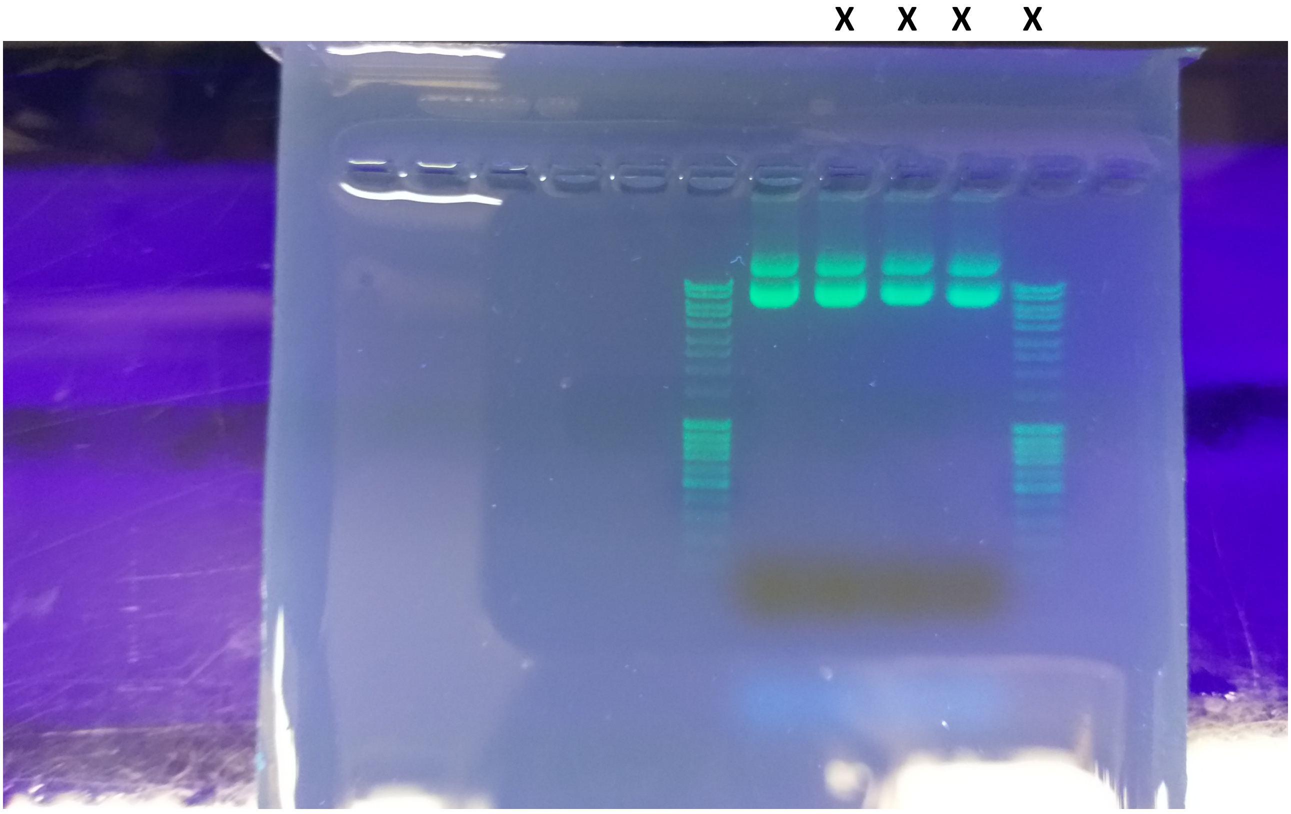

1b. Raw data used for Figure 3B
(the first two lines were used; X = lane not used in the Figure)

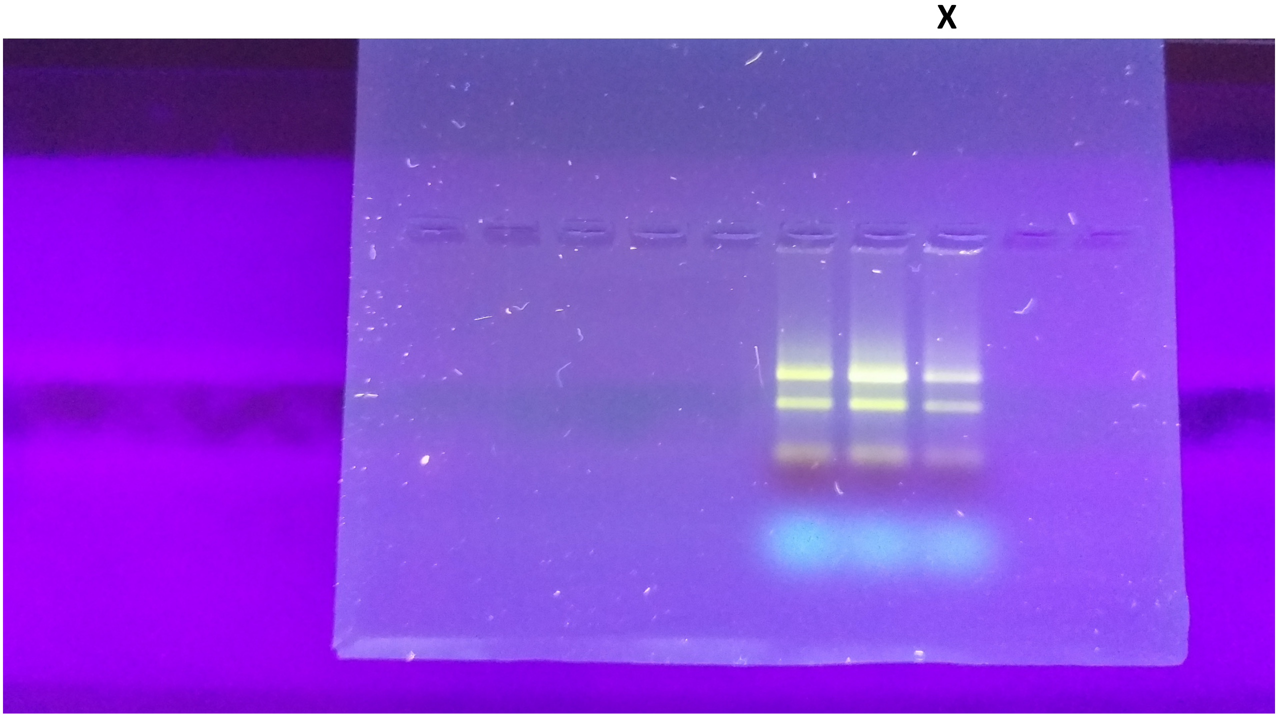

1b. Raw data used for Figure 3C

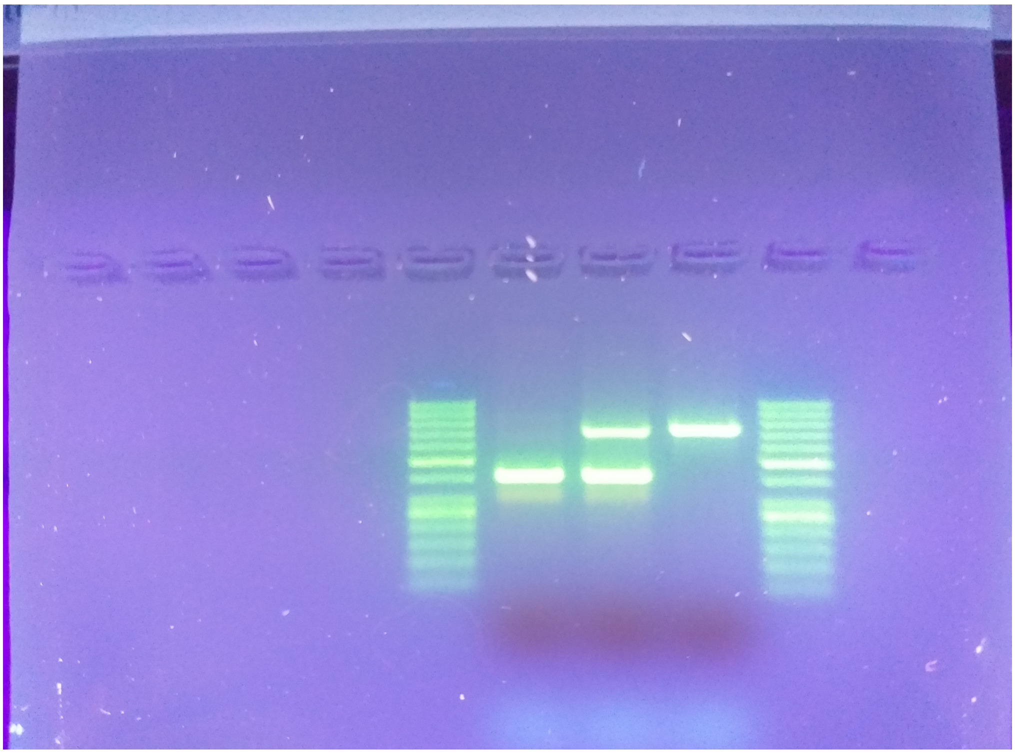

2. Raw uncropped images used to prepare Figure 4

2a. Figure 4

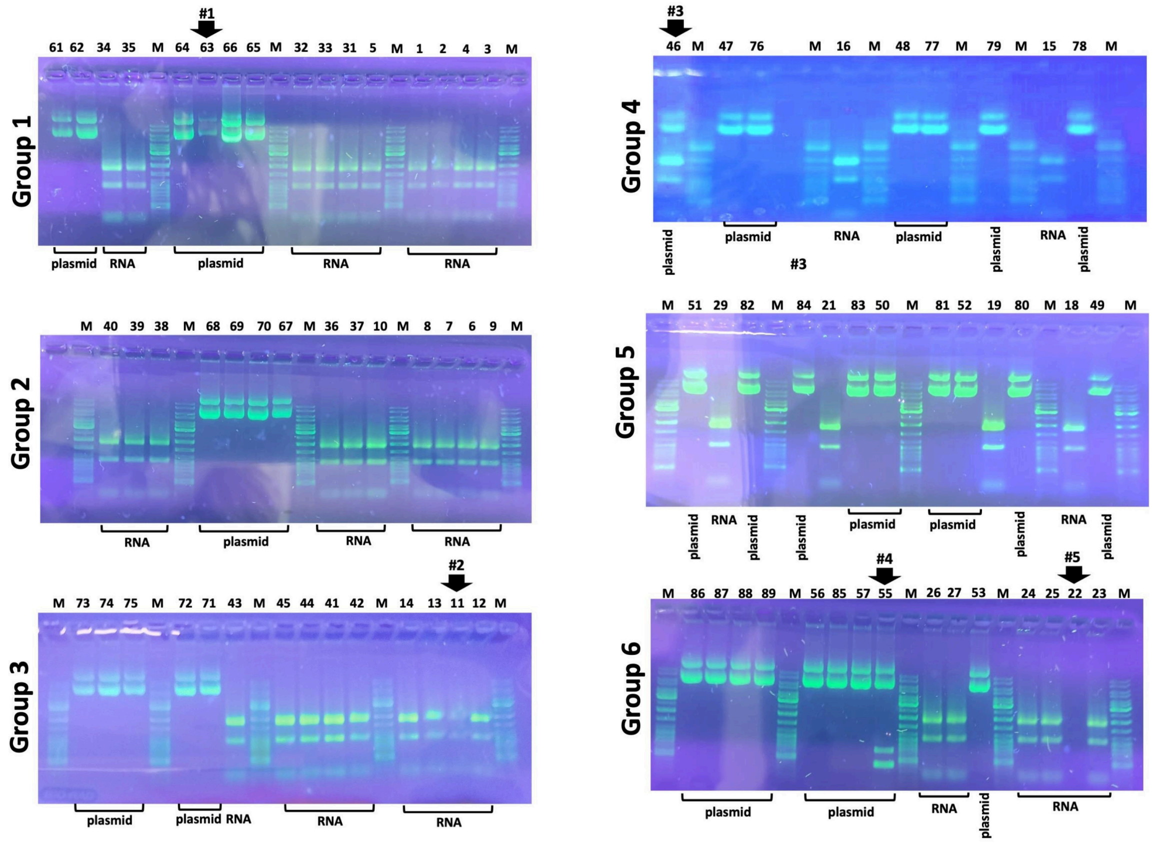

2b. Raw images of the gels

Group 1

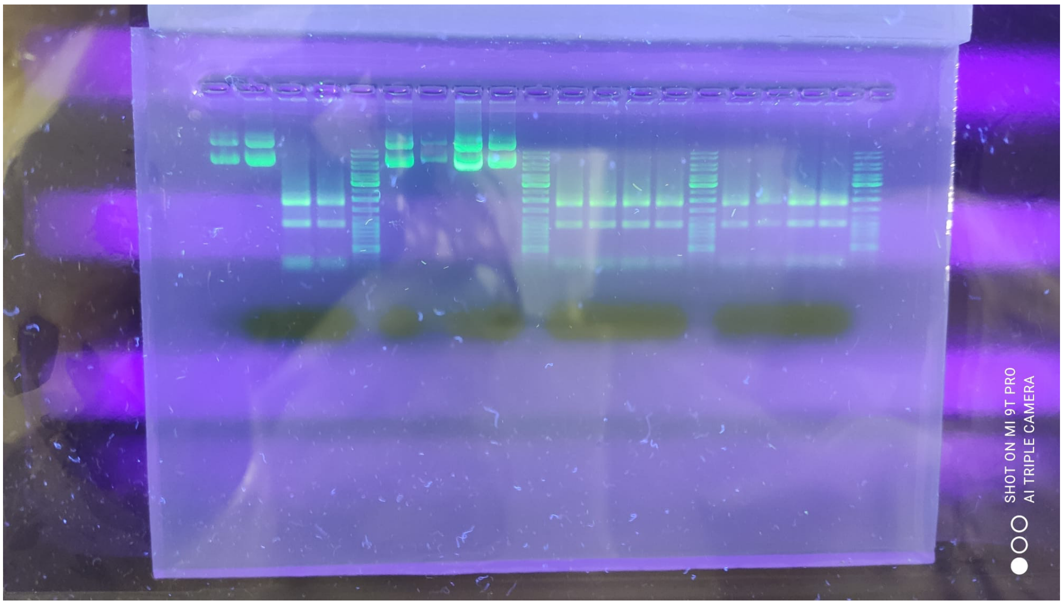

Group 2

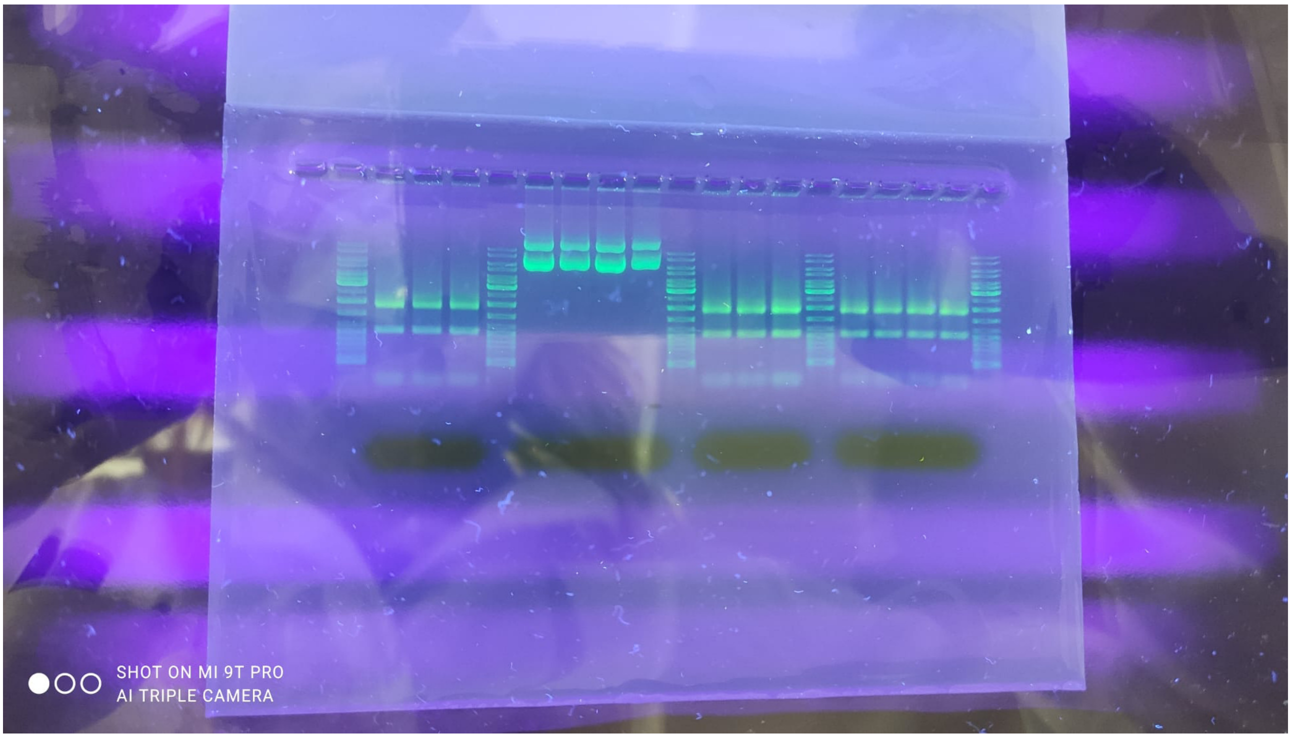

Group 3

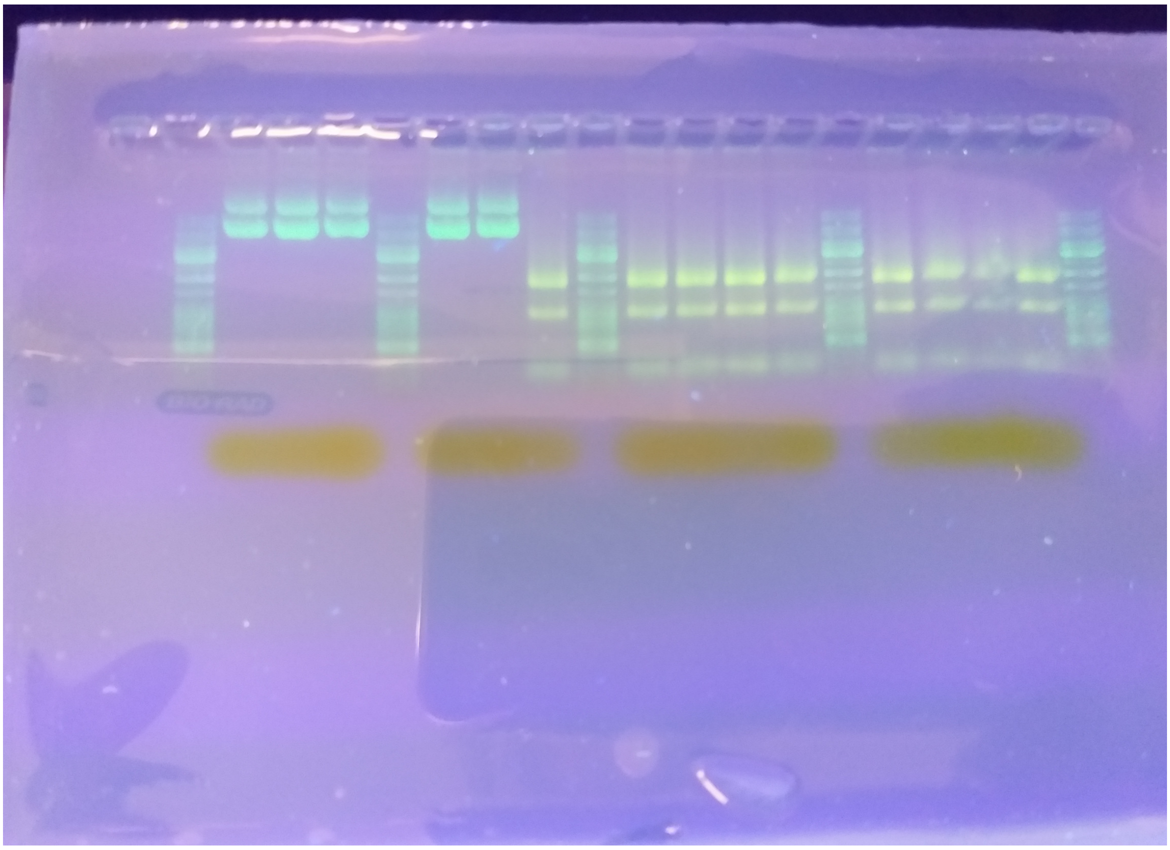

Group 4

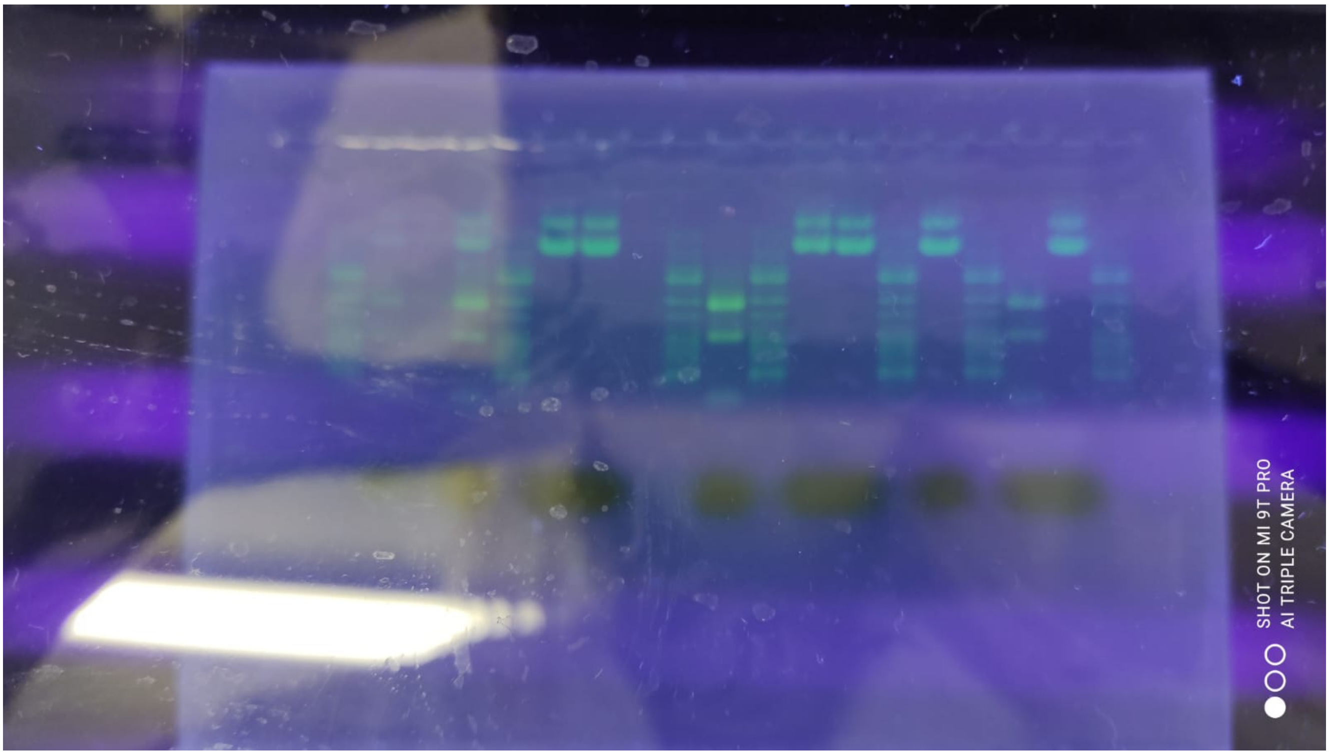

Group 5

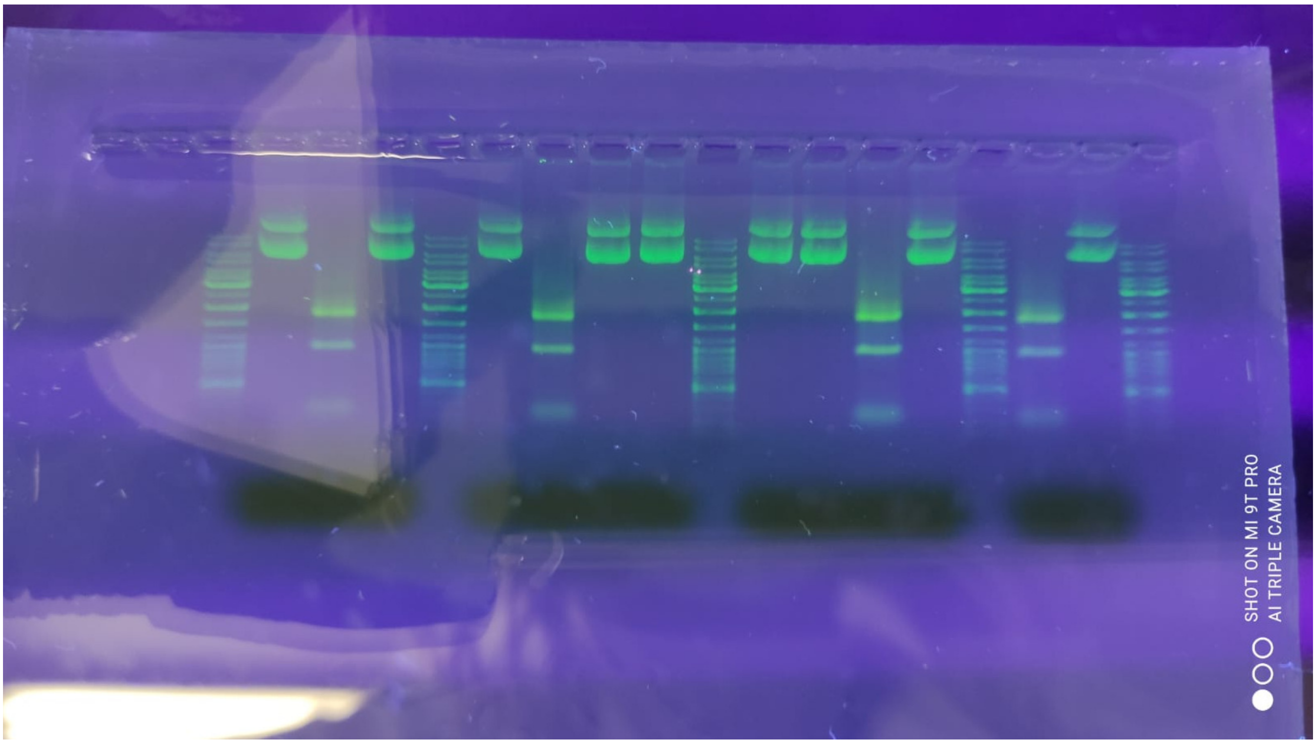

Group 6

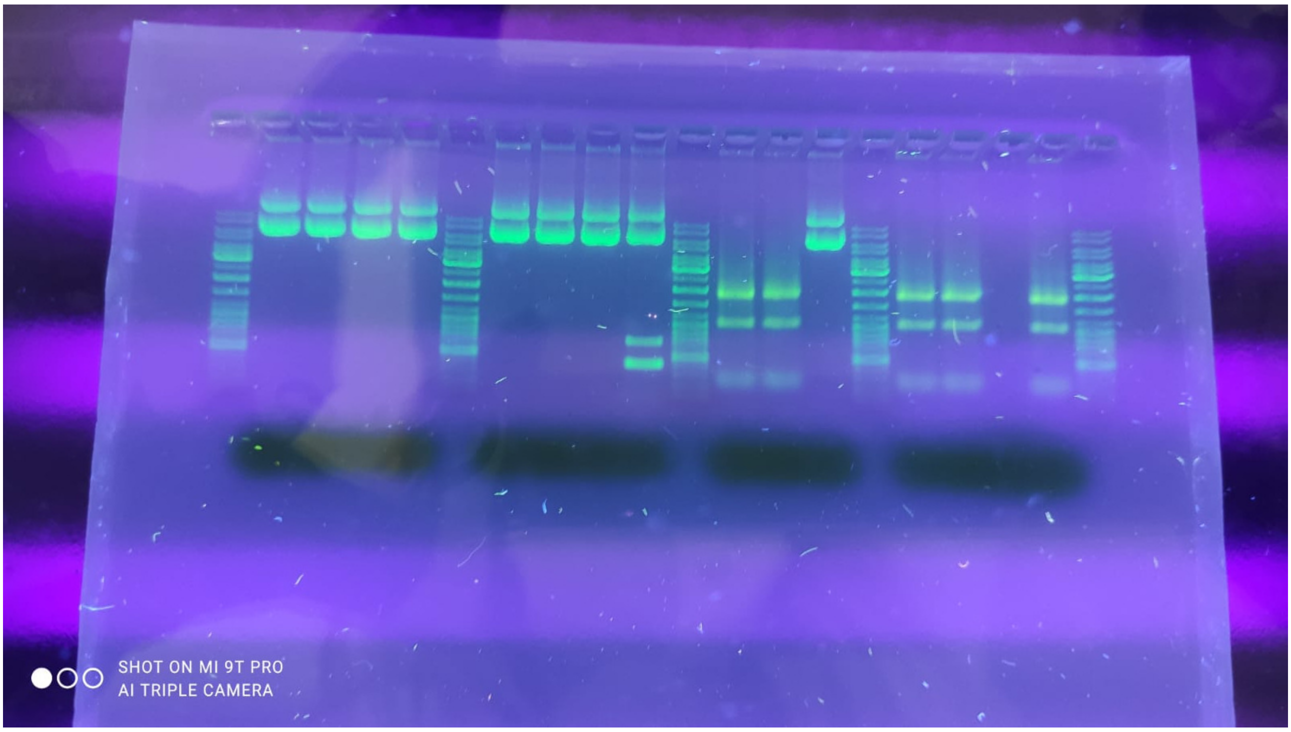

3. Raw uncropped images used to prepare Figure 5

3a. Figure 5

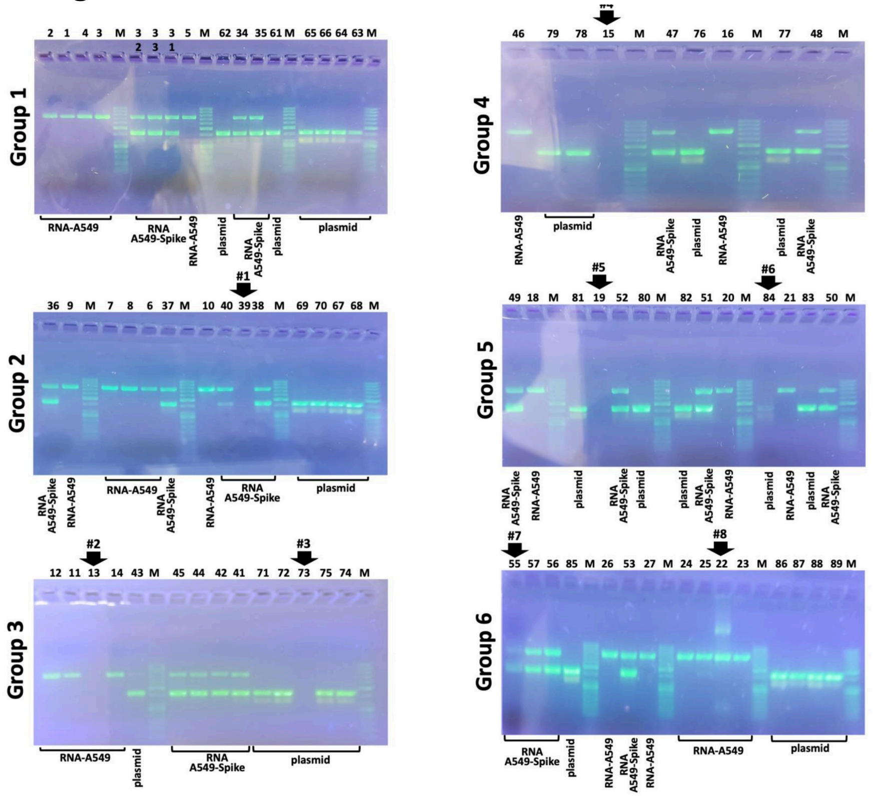

3b. Raw images of the gels

Group 1

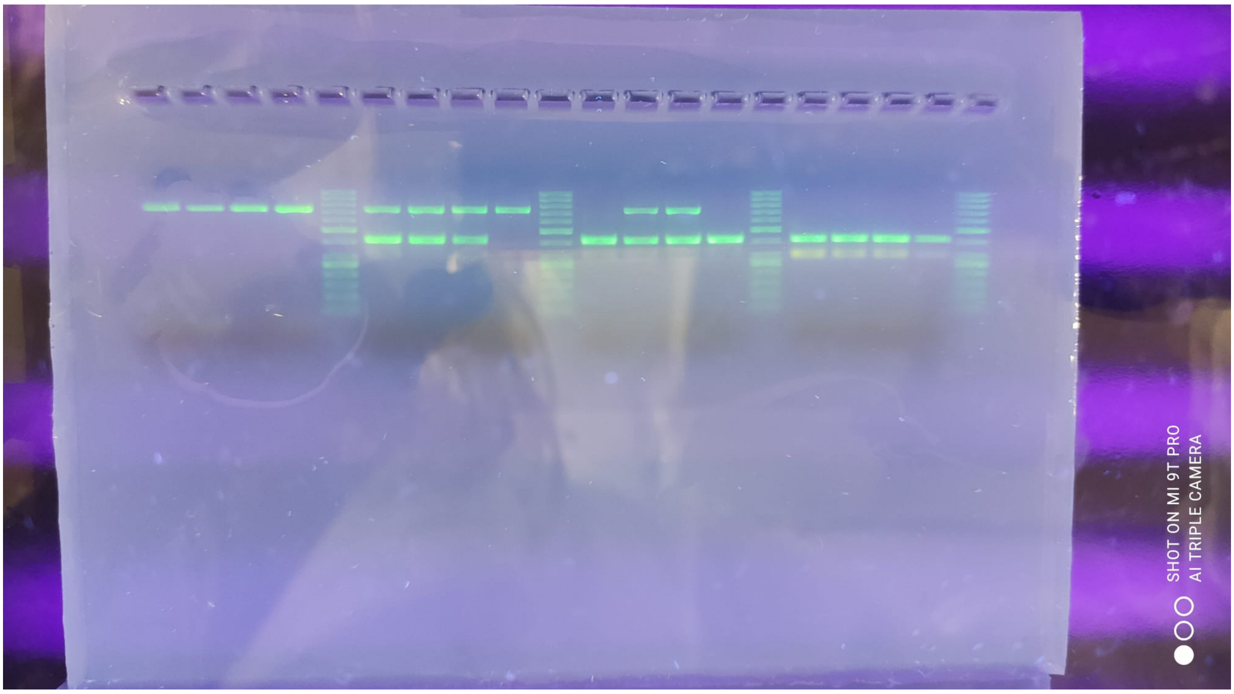

Group 2

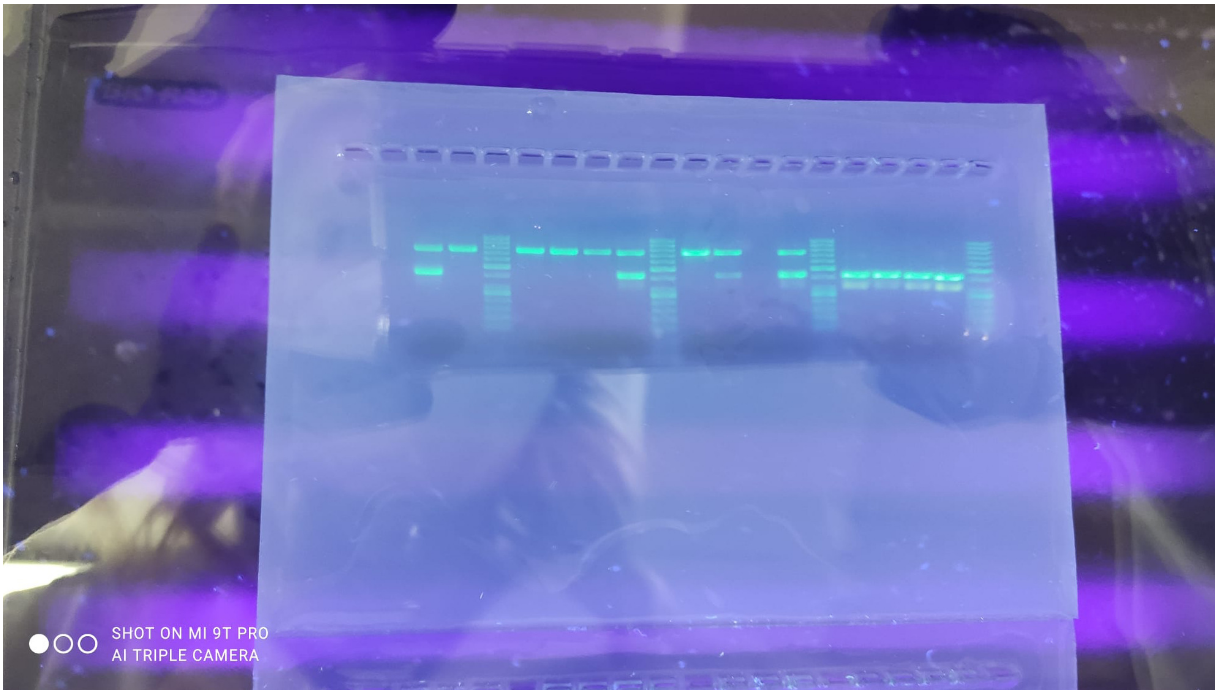

Group 3

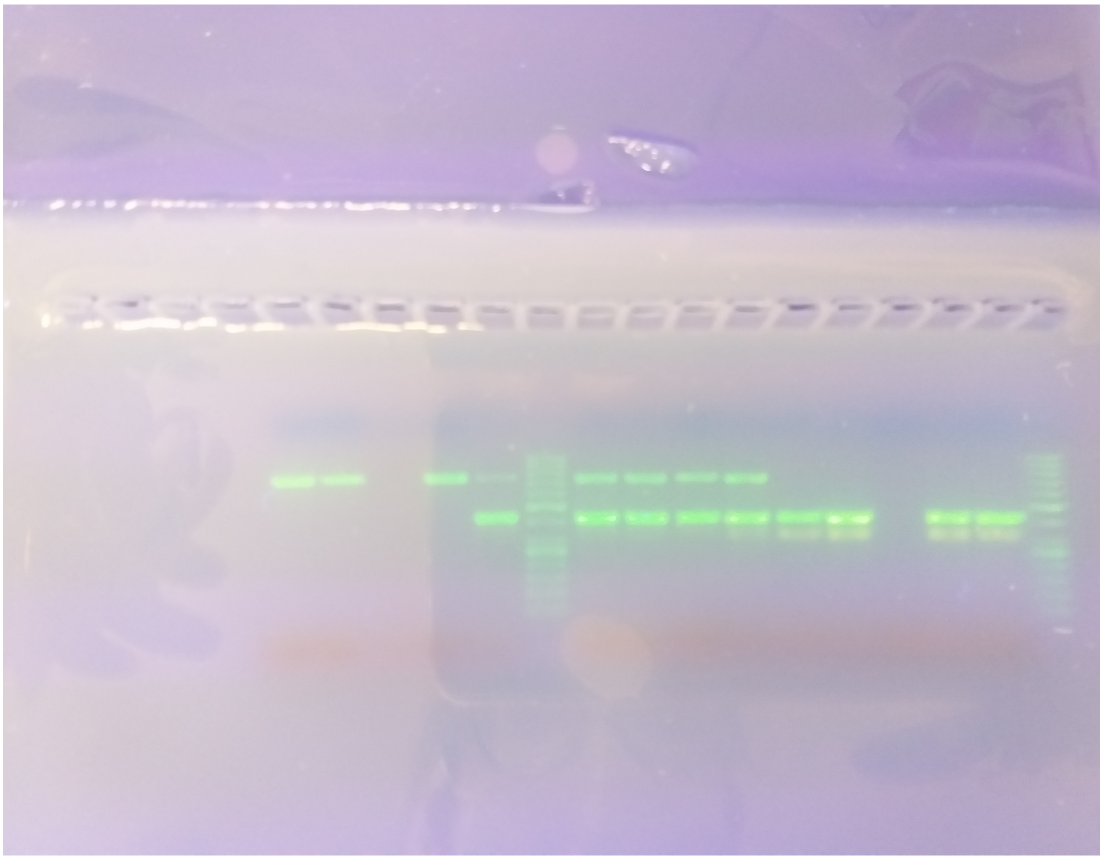

Group 4

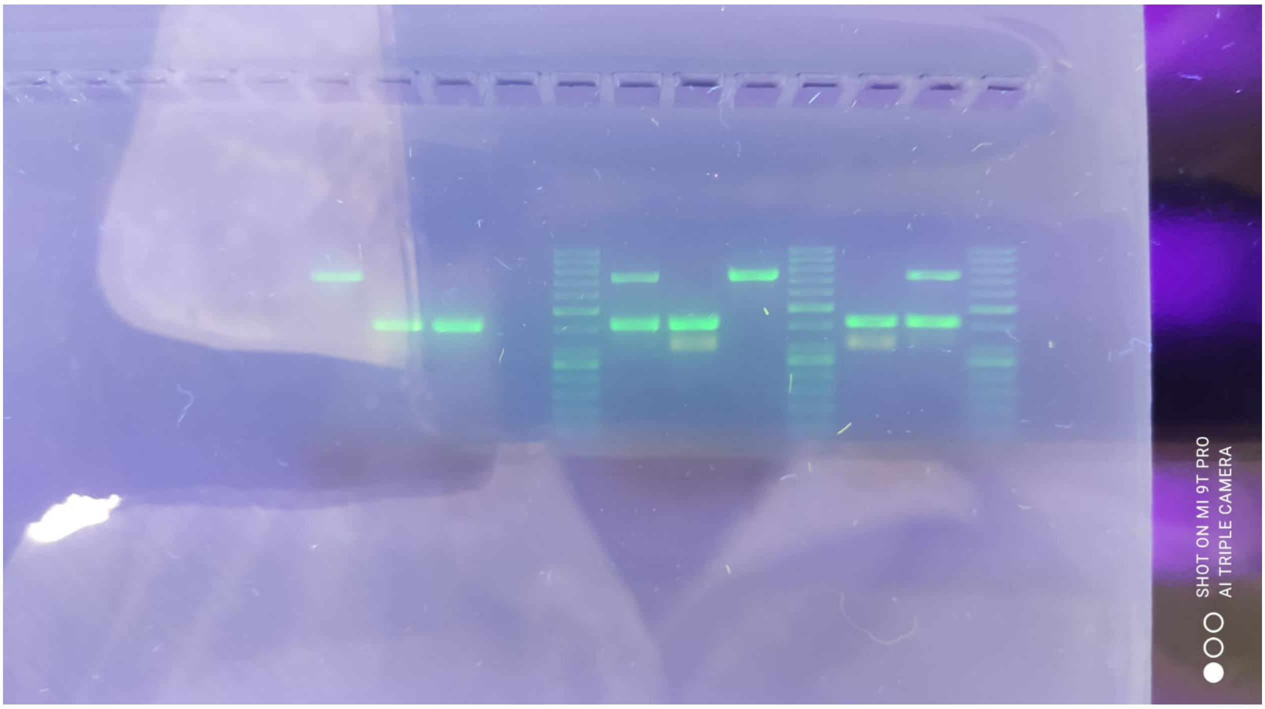

Group 5

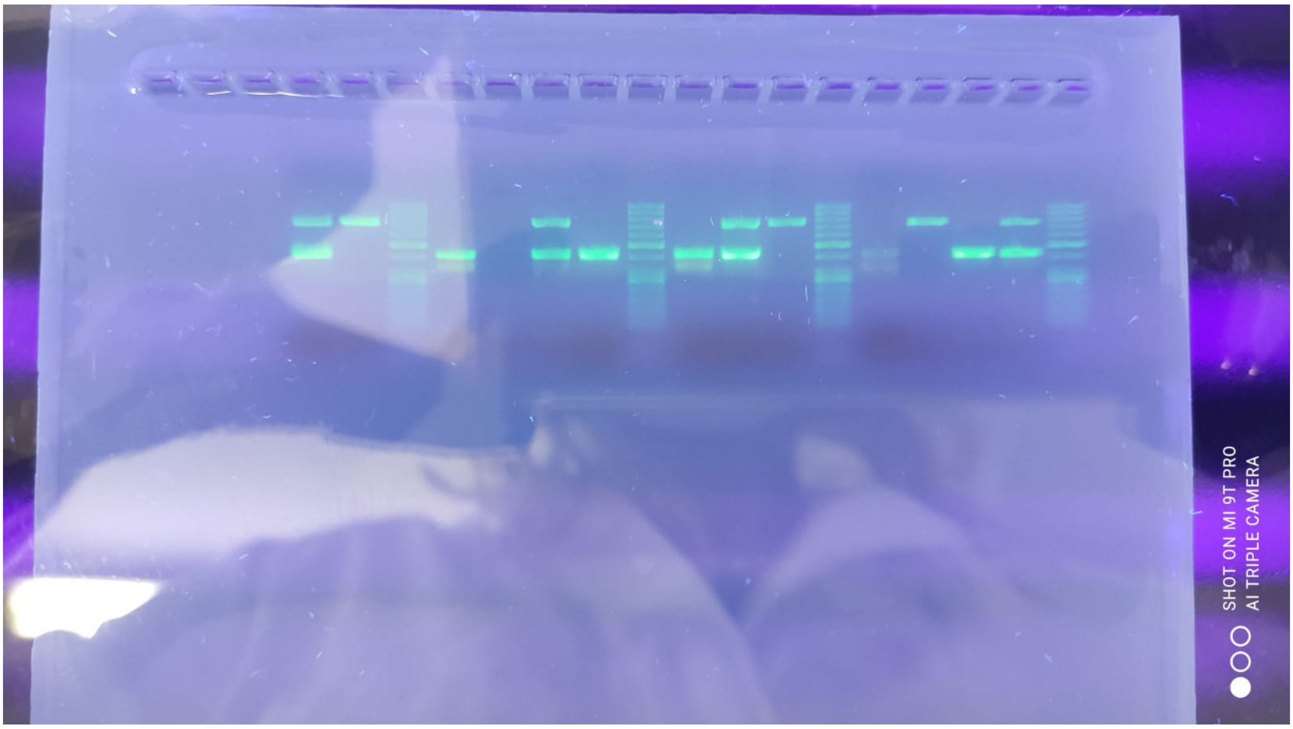

Group 6

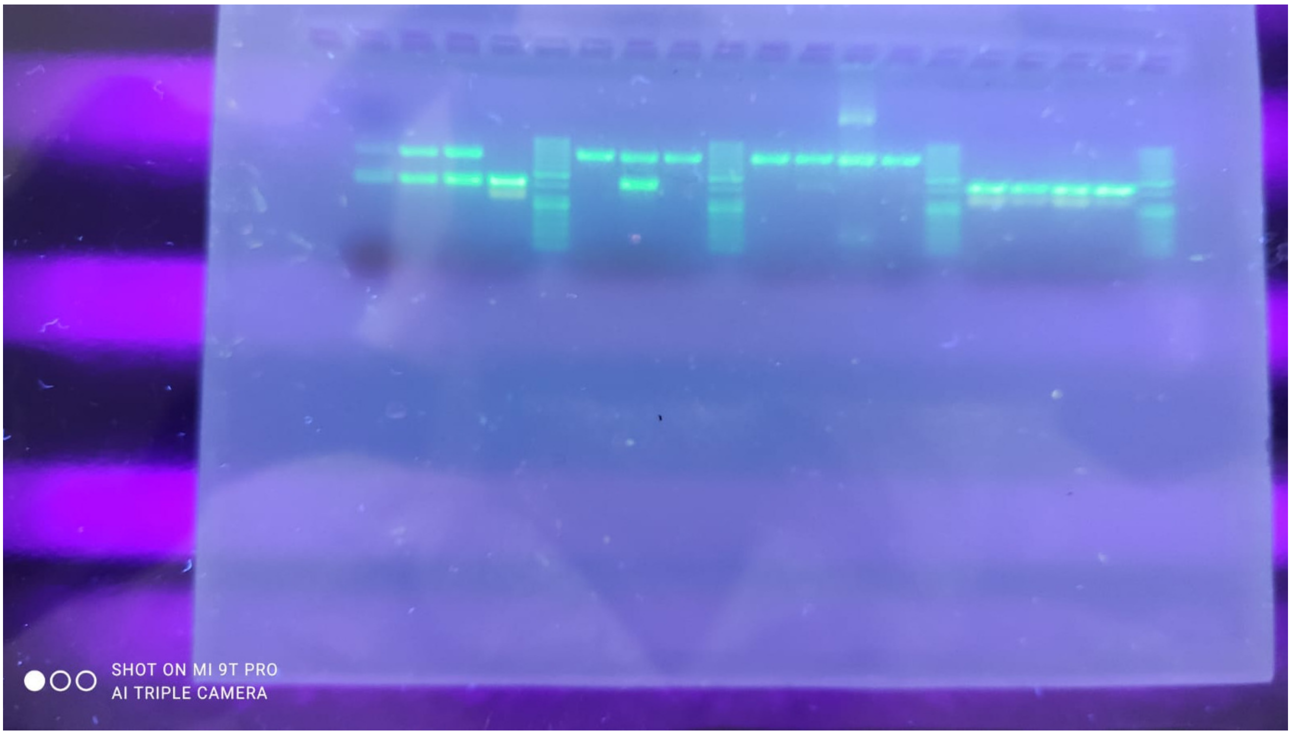

Comments.

1. Information on the loading order can be obtained after comparison with the relative Figures (Fig.3, Fig.4 and Fig.5)

2. The GelDoc System (Bio-Rad, Hercules, CA, USA) was used to capture the images.

3. The Molecular weight markers included were the following: (a) GeneRuler 50 bp DNA Ladder (Thermo Fisher Scientific, Walthman, Massachusetts, USA) and (b) GeneRuler 1 kb DNA Ladder (Thermo Fisher Scientific, Walthman, Massachusetts, USA).

4. The lanes not included in the final figures have been marked with an "X" above the lane on the original gel images.

5. All labeling and annotation have been performed without obscuring any data or background bands.
